# Integrating a soil temperature module into AquaCrop improves simulation of crop growth and yield under straw mulching

**DOI:** 10.3389/fpls.2026.1968615

**Published:** 2026-10-28

**Authors:** Yage Song, Fangyuan Zhang, Ye Wang, Ren Zhang

**Affiliations:** College of Engineering, Shenyang Agricultural University, Shenyang, China

**Keywords:** AquaCrop model, crop growth, model coupling, soil temperature, straw mulching

## Abstract

**Introduction:**

Straw mulching is a core practice in conservation agriculture, improving the soil hydrothermal environment, enhancing water-use efficiency, and increasing crop yield, and is widely regarded as an environmentally friendly and sustainable mulching technique. However, most existing crop models simplify or neglect mulch-induced regulation of soil temperature and water processes, thereby limiting their capacity for system-level assessment of crop growth and water dynamics under mulched conditions. This study aims to enhance the adaptability of AquaCrop to mulched environments and to evaluate the interactive effects of straw mulching on soil water–heat processes and crop growth.

**Methods:**

Based on the AquaCrop framework, a soil temperature submodule (ST) incorporating key surface-cover environmental factors was developed to extend the model’s capability to simulate soil temperature dynamics. Early-season crop development was driven by cumulative thermal time in the topsoil rather than air-temperature growing degree days, yielding a coupled model, AquaCrop–ST. A maize conservation-agriculture field experiment was conducted in Northeast China using locally prevalent mulching practices, with four treatments: straw crushing mulch, straw strip mulch, whole-straw mulch, and a no-mulch control. The coupled model was evaluated against observations.

**Results and discussion:**

Straw mulching significantly moderated topsoil temperature dynamics, lowering soil temperature by 1.77–4.46 °C during warm periods and reducing early-season thermal accumulation by 10–24%. The improved model reproduced topsoil temperature dynamics under mulching well, with R^2^ values of 0.92–0.95, RMSE values of 0.93–1.06 °C, and NSE values of 0.88–0.94, and also captured the seasonal patterns of deeper soil temperature. Compared with the original model, canopy development simulations were substantially improved, with RMSE decreasing from 33.49–33.73% to 3.13–8.13%. The improved model also successfully captured mulch-induced phenological delay and improved the simulation of soil water dynamics and evapotranspiration.

## 1 Introduction

Straw mulching, as an important conservation tillage practice, has been widely applied in dryland agriculture and the production of major field crops such as maize, wheat, and soybean (Busari et al., 2015). Northeast China is a major spring-maize production region characterized by typical rainfed agriculture and large fluctuations in soil hydrothermal conditions. Conservation tillage based on straw retention and reduced or no tillage has been widely promoted in this region, with the promoted area exceeding 3.3 × 10^6^ ha across the four northeastern provinces (Sui and Dai, 2025). Straw mulching regulates energy and water exchange within the soil–crop system, improves the root-zone hydrothermal environment, and consequently influences crop growth and productivity (Zhao et al., 2019).

Studies have shown that mulch layers can significantly alter the surface energy distribution (Alletto et al., 2011), reducing direct solar radiation and weakening wind speed effects, which in turn mitigate soil thermal fluctuations caused by extreme high or low temperatures (Chen et al., 2007). During spring and seedling stages, straw mulching often exhibits a certain insulating effect, while in summer it can effectively lower surface temperatures and alleviate heat stress (Kader et al., 2017). In terms of water management, straw mulching reduces soil evaporation (Dahiya et al., 2007), promotes rainfall infiltration, and enhances soil water-holding capacity (Liu et al., 2023), significantly improving water use efficiency under drought conditions. Numerous field trials have demonstrated that soil moisture content in the surface layer under mulched treatments is generally higher than that under bare soil, with more pronounced differences during dry periods (Wang et al., 2023). In the long term, straw mulching can increase soil organic matter, improve soil structure, promote microbial activity, and enhance nutrient cycling (Qin et al., 2024; Ninkuu et al., 2025), thereby providing a better habitat for crop roots. At the physiological level, root-zone water stress restricts transpiration, affecting dry matter accumulation and distribution (Liu et al., 2016). Additionally, soil temperature, as an important indicator of thermal conditions, directly influences crop phenology, canopy development, and yield formation (Stone et al., 1999). These hydrothermal improvements can promote coordinated root and shoot growth and enhance crop productivity (Qin et al., 2015). For example, compared with conventional tillage, different straw-mulching practices increased aboveground biomass by 1.56–5.48%, root mass density by 15.10–32.36%, root–shoot ratio by 10.38–32.74%, and maize grain yield by 3.79–12.40% (Wang et al., 2014). More recently, a three-year field experiment showed that straw mulching improved root–shoot coordination and increased maize grain yield by 8.2% relative to the non-mulched treatment (Liao et al., 2025).

In recent years, many crop models with varying physiological mechanisms, complexity, and application scales have been developed (Di Paola et al., 2016). Due to a lack of quantitative methods and field measurement data, most soil-crop system models are unable to simulate the effects of mulching on soil heat transfer, water movement, and crop growth (Liang et al., 2017). As a result, some studies have attempted to modify general crop models to quantify the impacts of mulching measures. For example, (Shen et al., 2021) incorporated the “membrane micropore effect” and a temperature compensation mechanism into the DSSAT-CERES model to improve simulations under plastic film mulching; (Jia et al., 2024) included the dynamic process of degradable films in DSSAT, enhancing evaporation and soil temperature simulations; (Chaves et al., 2024) proposed a dynamic parameterization scheme for crop residue cover quality, thickness, moisture, and thermal properties in STICS, but overall, these approaches remain predominantly empirical. APSIM provides a more process-based representation of crop residues, with SurfaceOM dynamically simulating residue mass, cover, and decomposition and SoilTemp simulating soil thermal dynamics (Balwinder-Singh et al., 2011; Cichota et al., 2021). However, crop phenology remains primarily driven by air-temperature-based thermal time, so mulch-induced changes in seed-zone temperature are not directly transferred to crop development (Yadav et al., 2025). More physically based attempts include the development of a film submodule in RZ-SHAW to explicitly solve energy and water fluxes beneath the membrane (Chu et al., 2022); Zhao et al. (2020) improved SWAP by revising the rainfall interception, evaporation, soil temperature, and crop growth modules to provide a more comprehensive description of hydrothermal processes under mulching.

Overall, these studies indicate that explicitly incorporating the thermal and dynamic properties of mulching materials into crop models helps to improve simulation accuracy under mulching scenarios. Empirical parameterization methods are simple and suitable for data-limited conditions, but they are still insufficient in reflecting the true physical processes of the mulch layer. High-physical models can describe energy and water transfer more accurately but are highly dependent on input data and parameters, limiting their application range. Notably, most of the aforementioned improvements focus on plastic film mulching, while system simulation studies on straw mulching remain relatively limited.

In this context, the AquaCrop model, due to its simplicity and low parameter requirements, has been widely used in farmland management and climate change research (Raes et al., 2009; Steduto et al., 2009), and is often used to simulate the effects of mulching on crop growth (Ćosić et al., 2017; Ran et al., 2017; Guo et al., 2018; Tan et al., 2018). Although AquaCrop has been widely applied under mulched field management conditions, its standard framework accounts for the suppression of soil evaporation by adjusting the soil evaporation coefficient according to mulch coverage and mulch type (Raes et al., 2009). Meanwhile, the original model lacks an independent soil temperature module and therefore cannot simulate the dynamic changes in soil temperature induced by straw mulching and their effects on crop growth and development. As AquaCrop has been increasingly applied under mulching management conditions, this limitation has received growing attention (Cheng et al., 2022; Feng et al., 2022; Zhao et al., 2023a), and researchers have begun to explore approaches to address it. For example, Mahmoud et al. (2022) modified AquaCrop-OS by converting the difference between soil and air temperatures under mulching into additional heat units during specific crop-growth stages. Similarly, Zhang et al. (2023) incorporated a temperature-compensation approach into AquaCrop to account for the warming effect of plastic-film mulching on maize development and improve the simulation of biomass and grain yield. More recently, Liu et al. (2025) enhanced AquaCrop by introducing stage-specific temperature-increase compensation coefficients for different plastic-film mulching treatments, which improved the simulation accuracy of canopy cover and aboveground biomass. However, these approaches mainly rely on empirically derived fixed temperature-compensation relationships, overlooking the dynamic variations in environmental temperature and being sensitive to the estimation of compensation coefficients, which may introduce additional uncertainty. Therefore, they do not fully address the lack of an explicit soil temperature–crop growth and development response process in AquaCrop.

In contrast, the soil temperature module in the hydrological model SWAT uses a lag-damping depth method and adjusts temperature fluctuations through a surface mulching weight factor, effectively capturing daily-scale soil temperature dynamics with low parameter requirements (Qi et al., 2019). This approach provides an important reference for constructing a soil temperature submodel suitable for straw mulching scenarios within the AquaCrop framework while balancing physical realism and computational simplicity.

To address the lack of an explicit soil temperature–crop growth and development response process in AquaCrop, this study aims to enhance and extend the model, thereby improving its capability to simulate crop development and yield formation under straw mulching in conservation tillage systems. The specific objectives are as follows:(1) Develop a soil temperature submodule suitable for straw mulching and integrate it into the AquaCrop framework to realize daily-scale synchronous simulation of soil temperature and crop growth;(2) Calibrate and validate the model’s performance in simulating soil temperature, canopy development, and yield using field experimental data from maize conservation tillage fields in Northeast China;(3) Elucidate the mechanisms by which different mulching practices in conservation tillage regulate soil hydrothermal conditions to modulate crop growth.

## 2 Materials and methods

### 2.1 Study area

The experiment was conducted at the Fuxin Conservation Tillage Experimental Station in Liaoning Province, China (42°1′N, 121°40′E, elevation 219 m), as shown in **Figure 1**. The station is located in the northeastern plain, a typical rain-fed dryland agricultural area with a semi-arid climate. During the 2024 maize growing season, the average temperature was 22.8 °C, and the total accumulated precipitation was 560.2 mm. Precipitation was unevenly distributed and mainly concentrated in July and August (**Figure 1c**). The soil texture in the 0–20 cm layer is loam, and in the 20–100 cm layer, it is silty loam. Detailed soil properties are shown in **Table 1**. The region typically adopts no-tillage with straw mulching as a conservation tillage practice (Tang et al., 2011). In this experiment, straw mulching began after the harvest in the previous fall, and the 2024 growing season was the first year of the straw mulching treatment.

**Figure 1:**
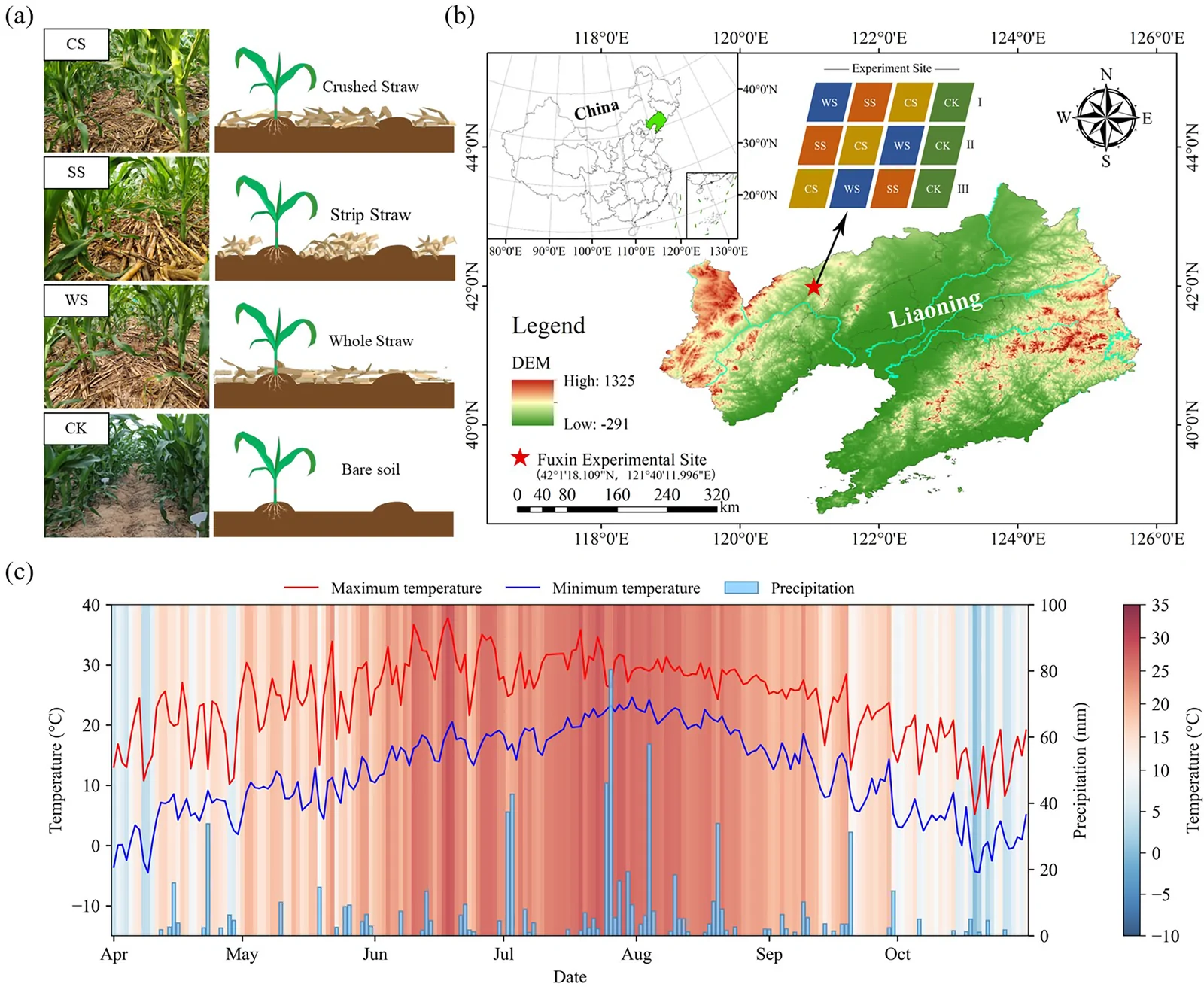
Geographic location of the experimental site and treatment setup. **(a)** Four mulching treatments applied in this study; **(b)** geographic location of the experimental site and experimental plot layout; **(c)** dynamic meteorological variables during the experiment.

**Table 1:** Soil physical and hydraulic parameters measured at the study site: texture (sand, silt, and clay content), bulk density (*ρb*), soil organic matter (OM), saturated water content (*θ_sat_*), field capacity (*θ_FC_*), permanent wilting point (*θ_PWP_*), and saturated hydraulic conductivity (*K_sat_*).

Depth (cm)	Sand (%)	Silt (%)	Clay (%)	OM (g kg^-1^)	*ρ_b_* (g cm^−3^)	*θ_sat_* (cm^3^ cm^−3^)	*θ_FC_* (cm^3^ cm^−3^)	*θ_PWP_* (cm^3^ cm^−3^)	*K_sat_* (cm d^−1^)
0–20	40	40	20	8.74	1.57	0.419	0.271	0.13	22
20–40	33	64	3	5.07	1.45	0.452	0.282	0.10	50
40–60	34	64	2	3.41	1.42	0.464	0.296	0.09	49
60–80	38	59	3	3.76	1.43	0.460	0.300	0.10	52
80–100	38	55	7	1.11	1.47	0.445	0.286	0.11	48

### 2.2 Experimental design

The field experiment included four locally used tillage treatments: chopped straw mulching (CS), strip straw mulching (SS), whole straw mulching (WS), and a no-mulching control (CK). Each treatment had three replicates, resulting in 12 experimental plots, each measuring 105 m^2^ (7 × 15 m). The CS, SS, and WS treatments were randomly arranged within each replicate, whereas CK plots were positioned at fixed locations to facilitate consistent comparisons and uniform field management. This layout also avoids the risk of straw transfer from mulched plots to the control CK plots. Guard rows were established around the experimental area to mitigate potential edge effects. The specific implementation details of each treatment are presented in **Table 2**.

**Table 2:** Field plot experimental treatment setup.

Treatment	Name	Implementation method
CS	No-till chopped straw mulching	After autumn harvest, maize stalks are chopped (≤10 cm) and evenly mulched on the soil surface
SS	No-till strip straw mulching	After autumn harvest, whole maize stalks are evenly mulched along the ridge furrow
WS	No-till whole straw mulching	After autumn harvest, whole maize stalks are mulched on the soil surface perpendicular to the ridge direction
CK	No-till no mulching	After autumn harvest, surface stalks are removed

The maize cultivar used in this study was Xiyuan 1908, a medium- to late-maturing spring maize hybrid adapted to Northeast China, planted at a row spacing of 60 cm and a plant spacing of 30 cm. Under spring-sown conditions, the cultivar has a growth duration of approximately 131 d from emergence to maturity and requires an active accumulated temperature (daily mean temperature ≥10 °C) of approximately 2800 °C d. Mature plants typically produce approximately 22 leaves. The kernels are semi-dent, with a 100-kernel weight of approximately 45.4 g, and the cultivar is representative of locally cultivated maize varieties. The straw mulch rate was 7500 kg hm^−2^ (Yuan et al., 2024). Base fertilizer was applied once with sowing: urea (N 46.4%) at 200 kg ha^−1^ and diammonium phosphate (P_2_O_5_ 46%, N 18%) at 250 kg ha^−1^. The experiment relied on natural precipitation with no-tillage and no irrigation throughout the entire growth period. Field management followed local agricultural production technical standards.

### 2.3 Observation indicators and methods

Meteorological data required by the AquaCrop model were obtained from the Fuxin meteorological station near the experimental site. Soil temperature and soil moisture were monitored in real-time using GS1-AL4G1RS IoT data loggers (Dalian Cloud Power Technology Co., Ltd., China). Sensors (UB-STH-N1, based on frequency domain reflectometry) were installed at a depth of 10 cm in the plow layer, collecting data every hour and automatically uploading it to the cloud platform via a 4G network. All sensors were calibrated before installation.

Additionally, during each crop growth stage, soil samples from 0–100 cm depth (at 20 cm intervals) were collected using a soil auger. The 0–100 cm soil profile was selected to encompass the main maize rooting zone and associated soil-water dynamics, as most maize roots are concentrated within the upper 60 cm (Zhou and Zhao, 2019), while this depth is also consistent with the effective root-zone scale represented in AquaCrop (Steduto et al., 2009). Soil temperature at each depth was measured using a portable soil thermometer. Soil samples were oven-dried at 105°C for 24 hours and weighed to calculate the gravimetric water content, which was then converted to volumetric water content. A portable soil thermometer was used to obtain temperature measurements across the entire soil profile for model evaluation. In addition, these portable measurements provided an independent check of the continuously monitored data and helped identify potential sensor drift during long-term field operation.

During critical maize growth stages (V3, V6, VT, R3, and R6), crop growth indicators were measured. In the AquaCrop model, crop leaf growth and development are represented by the Green Canopy Cover (CC) rather than the Leaf Area Index (LAI) (Steduto et al., 2009). Since LAI is generally easier to obtain in field observations, this study measured the length and maximum width of fully expanded leaves of three maize plants in each plot to calculate individual plant LAI, which was then converted to CC using the empirical formulas given in Equations 1 and 2 (Zhang et al., 2021):

(1) $$LAI=1.0\times 10^{-8}\times \rho \times \sum_{k=1}^{n}\left(0.75\times L_{k}\times W_{k}\right)$$

(2) $$\;CC=1.005\times {\left[1-\text{exp}\left(-0.6LAI\right)\right]}^{1.2}$$

where *L_k_* is the length of the k-th maize leaf (cm), *W_k_* is its maximum width (cm), 0.75 is the leaf shape correction factor, and *ρ* is the plant density (plants ha^−1^).

The LAI–CC relationship adopted in this study has been evaluated in previous maize studies. Hsiao et al. (2009) reported high agreement between simulated and observed canopy development in the original AquaCrop maize parameterization, with a Willmott index of agreement of ≥0.98 in most cases. Nielsen et al. (2012) independently evaluated the LAI–CC relationship for maize under dryland and limited-irrigation conditions and confirmed a strong nonlinear relationship between LAI and canopy cover. The same LAI–CC relationship has also been subsequently applied in AquaCrop-based maize studies under different growing and water-management conditions (Heng et al., 2009; Lu et al., 2021; Shan et al., 2022).

At maize physiological maturity, border rows were excluded to minimize edge effects, and a representative area of 35 m^2^ in the center of each plot was manually harvested for grain yield determination. Twenty representative ears were randomly selected from each treatment to determine yield components, including kernel number per ear and 100-kernel weight. Grain yield was adjusted to a standard moisture content of 14% and expressed as t ha^−1^.

### 2.4 AquaCrop model description

The AquaCrop model, developed by the Food and Agriculture Organization (FAO), is a water-driven crop model designed to quantify crop responses to different water conditions (Raes et al., 2009; Steduto et al., 2009). AquaCrop expresses crop development through green CC and separates crop evapotranspiration (ETc) into crop transpiration (Tr) and soil evaporation (E). Crop transpiration is determined from reference evapotranspiration (ETo), canopy cover, the crop transpiration coefficient, and water-stress effects. Aboveground biomass (B) is then calculated from cumulative crop transpiration using the normalized crop water productivity (WP*), and final yield (Y) is obtained from biomass through the harvest index (HI). The core relationships are shown in Equations 3–6:

(3) $$Tr=K_{s}CC^{*}K_{c\text{Tr},x}ET_{o}$$

(4) $$E=Kr\times Ke\times ET_{o}$$

(5) $$B=WP^{*}\sum \left(\frac{Tr}{ET_{o}}\right)$$

(6) $$Y=f_{HI}HI_{o}B$$

where *K_s_* represents the soil water stress coefficient, CC^*^ is the actual canopy cover (%) corrected for microenvironmental effects, $K_{cTr,x}$ is the maximum standard crop transpiration coefficient, *K_r_* is the evaporation reduction coefficient, which adjusts for the effects of surface water shortages, and *K_e_* is the soil evaporation coefficient. Additionally, *f_HI_* is the harvest index adjustment factor, and *HI_o_* is the reference harvest index.

By considering the interactions between soil and crops, field management practices such as mulching have been incorporated into AquaCrop (Raes et al., 2009). In the calculation of soil evaporation, AquaCrop assumes uniform distribution of the canopy and mulch in the field, using a one-dimensional parameterization method to adjust the soil evaporation coefficient *K_e_* for factors such as canopy shading effect $K_{e\left(adj,cc\right)}$, surface mulch (such as straw or plastic film) $K_{e\left(adj,m\right)}$, and local wetting effects due to irrigation $K_{e\left(adj,w\right)}$ as described in Equations 7–10:

(7) $$Ke=\left(\begin{array}{c} 1-CC^{*} \end{array}\right)Ke_{x}$$

(8) $$Ke_{adj,cc}=\left(1-f_{cc}CC_{top}\right)\left(1-CC^{*}\right)Ke_{x}$$

(9) $$Ke_{\text{adj, m}}=\left(1-f_{m}\frac{Percent\;covered\;by\;mulch\;}{100}\right)\left(1-{\text{CC}}^{*}\right)Ke_{x}$$

(10) $$Ke_{adj,w}=f_{w}\left(1-CC^{*}\right)Ke_{x}$$

where 1 – CC^*^ represents the adjusted proportion of the uncovered soil surface, while *K_e_*,*_x_* is the maximum soil evaporation coefficient for a fully wetted and unshaded soil surface. The coefficient *f_cc_* represents the shading effect of dead canopy cover, with values ranging from 0 to 1. The coefficient *f_m_* adjusts for the effect of mulch on soil evaporation, with a value of 0.5 for plant material mulches and close to 1.0 for plastic mulches. The proportion of soil surface covered by mulch is expressed as a percentage between 0 and 100%, and *f_w_* is an irrigation parameter, which is not applied when rainfall occurs on the same day.

### 2.5 AquaCrop-ST model description

In the AquaCrop daily-scale simulation, air temperature is the primary environmental variable driving crop development and water movement. It not only directly affects crop physiological processes but also indirectly influences the water balance of the entire system by regulating developmental processes, morphological development, and water demand. However, previous studies have shown that crops such as maize, in their early stages, have their meristematic tissue located underground, with the developmental rate primarily controlled by soil temperature rather than air temperature (Stone et al., 1999; Zhao et al., 2020). Straw mulching often reduces the surface soil temperature, and if air temperature continues to be used as the driving factor, it can lead to systematic errors in growth stage predictions (Hou et al., 2014). Considering that increasing long-term soil temperature observations would make data collection more difficult and limit model applications, process-based or empirical soil temperature models are able to simulate temperature dynamics with higher accuracy. Therefore, this study developed a soil temperature submodule (ST) based on the AquaCrop model, forming the AquaCrop-ST model, which extends the soil temperature simulation function. By using the surface soil temperature (Ts) simulated by the ST module, the model replaces air temperature to calculate effective accumulated temperature, thereby more accurately quantifying the regulation of soil temperature on early crop development under mulching conditions.

The coupling mechanism of the model is shown in **Figure 2**. In the AquaCrop-ST model, soil temperature is a key environmental variable that influences crop growth and water use processes through multiple mechanisms. The model first calculates soil temperature based on crop growth, soil moisture information, and surface mulching practices. Soil temperature determines the daily effective accumulated temperature accumulation rate, directly controlling phenological processes. Temperature reductions caused by mulching delay developmental stages, which in turn affect the rate, timing of maximum, and duration of CC expansion. The level of canopy development determines the crop transpiration (Tr) demand and water consumption, influencing the distribution of soil water evaporation. Additionally, temperature regulates root growth and distribution, directly affecting the soil’s water absorption capacity. Under mulching conditions, the temperature environment delays root expansion to some extent, while simultaneously increasing the availability of soil moisture. Furthermore, temperature stress inhibits key physiological processes (such as pollination), limiting biomass accumulation and yield formation. As biomass growth is determined by both Tr and WP*, straw mulching indirectly affects water use efficiency and final yield outcomes through its regulation of temperature.

**Figure 2:**
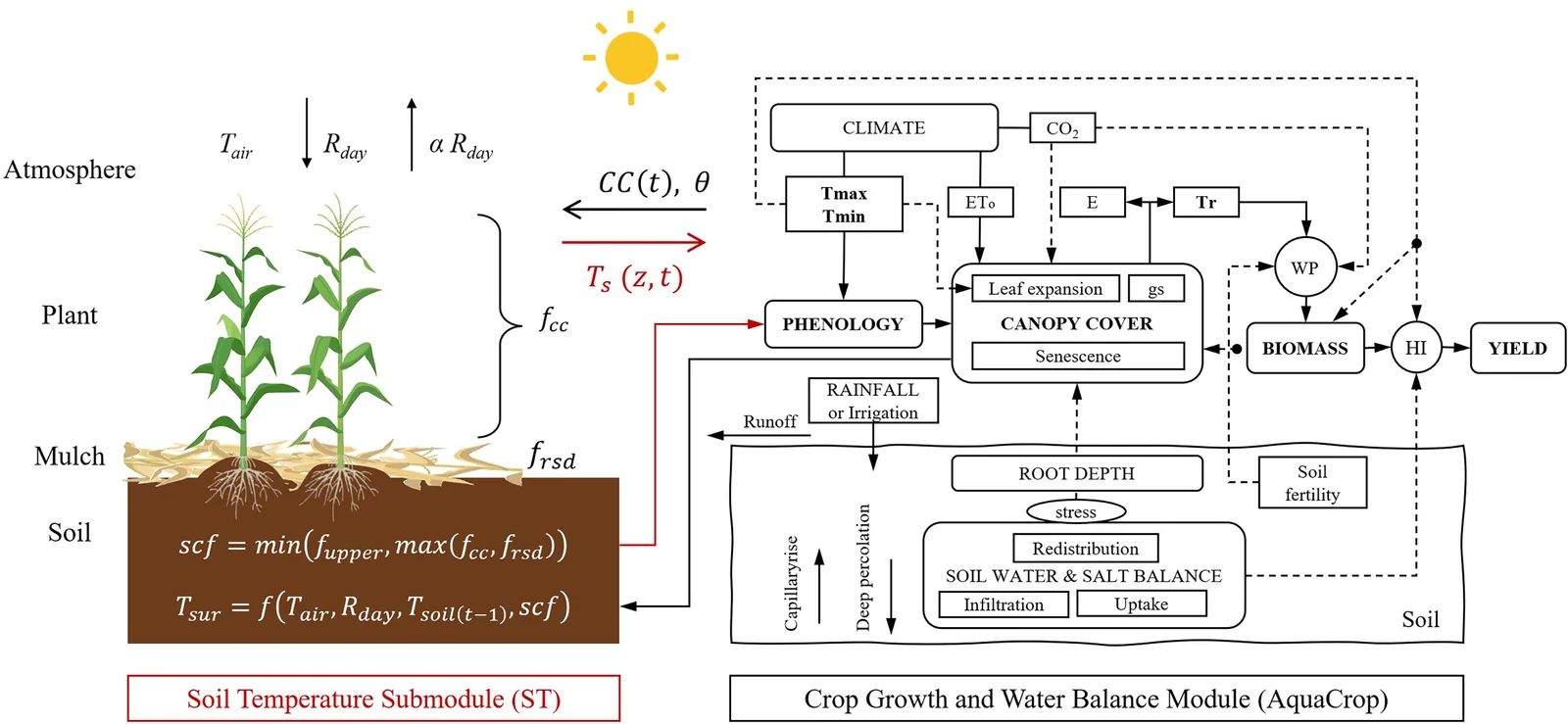
Coupling structure of the AquaCrop-ST model. The red box represents the newly developed ST module, while the black box represents the original AquaCrop model. The red lines and arrows represent the information flow from the ST module to AquaCrop. In the figure, *R_day_* represents solar radiation, *α* is the surface albedo, *f_cc_* is the canopy cover function, *f_rsd_* is the straw mulch function, *T_air_* is air temperature, and *T_max_* and *T_min_* are the maximum and minimum air temperatures, respectively. *g_s_* is the stomatal conductance. In the model structure, canopy cover *CC*(*t*) and soil moisture *θ* are passed from AquaCrop to the ST module. As the output of the ST module, soil temperature $T_{s}\left(z,t\right)$ feeds back into crop phenology and growth processes within AquaCrop. The AquaCrop framework on the right-hand side is adapted from Steduto et al. (2009).

The calculation process is shown in **Figure 3**. The model operates on a daily time step and uses Growing Degree Days (GDD) to quantify the impact of temperature on crop development. The process begins by reading daily meteorological data and checking various management practices to generate the management queue. Then, the previous day’s crop growth status is input into the soil temperature submodule to obtain the current day’s soil temperature. For straw mulching treatments, during the early crop development stages that are sensitive to temperature, *T_soil_* is used to replace air temperature to calculate accumulated temperature and update crop development status. In subsequent stages, the model returns to using standard air temperature as the driving factor. After updating for the day, the model moves to the next time step, repeating this process until the entire growing season ends. Through this mechanism, AquaCrop-ST can dynamically reflect the comprehensive impacts of soil temperature, under mulching conditions, on crop growth, water balance, and yield formation.

**Figure 3:**
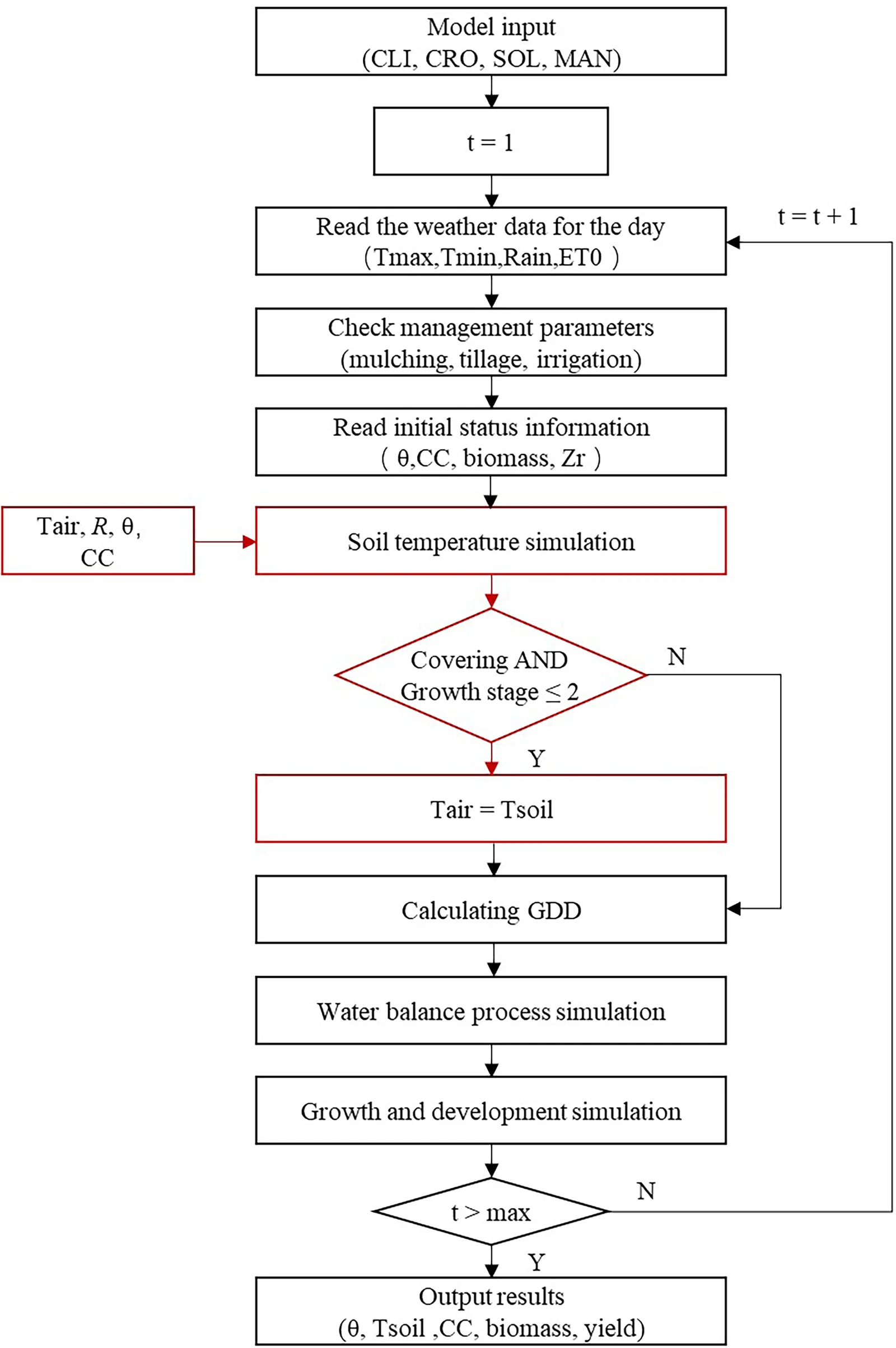
Calculation flowchart of the daily execution cycle of the AquaCrop-ST coupled model. *Z_r_* is the root depth, *T_soil_* is soil temperature. The red box indicates the ST module.

#### 2.5.1 Soil temperature submodule

In this study, we referenced and modified the SWAT soil temperature model (P. W. Gassman et al., 2007), which accounts for the lag effect, depth effect, and the impact of surface mulching during the calculation process. This model is capable of simulating the dynamic changes in surface and profile soil temperature more effectively. Based on the original recursive structure, we modified the surface temperature calculation to more accurately capture the regulation of soil thermal environment by straw mulching.

The daily average soil temperature at the center of each layer is calculated using Equation 11:

(11) $$T_{soil}\left(z,d_{n}\right)=\lambda T_{soil}\left(z,d_{n-1}\right)+\left(1-\lambda \right)\left[df\left({\bar{T}}_{Aair}-T_{sur}\right)+T_{sur}\right]$$

where $T_{\text{soil}}\left(z,d_{n}\right)$ is the soil temperature (°C) at depth *z* (mm) on day *d_n_*, λ is the lag coefficient, ranging from 0.0 to 1.0, used to control the influence of the previous day’s temperature on the current temperature. SWAT sets this value at 0.80. $T_{\text{soil}}\left(z,d_{n-1}\right)$ is the soil temperature (°C) at the same depth on the previous day, *d_f_* is the depth factor, used to quantify the influence of soil depth below the surface on soil temperature, ${\bar{T}}_{\text{Aair}}$ is the annual average air temperature (°C), and *T*_sur_ is the surface soil temperature (°C) on the given day.

The depth factor *d_f_* and its associated variables are calculated using Equations 12–16:

(12) $$df=\frac{zd}{zd+\text{exp}\left(-0.867-2.078zd\right)}$$

(13) $$zd=\frac{z}{dd}$$

(14) $$dd=dd_{max}exp\left[\text{ln}\left(\frac{500}{dd_{max}}\right){\left(\frac{1-\varphi }{1+\varphi }\right)}^{2}\right]$$

(15) $$dd_{max}=1\times 10^{3}+\frac{2500\rho_{b}}{\rho_{b}+686exp\left(-5.63\rho_{b}\right)}$$

(16) $$\varphi =\frac{SW}{\left(0.356-0.144\rho_{b}\right)z_{tot}}$$

where *zd* is the ratio of the soil layer’s center depth to the damping depth, *dd* is the damping depth (mm), *dd_max_* is the maximum damping depth (mm), *SW* is the soil profile water content, expressed as the water depth in the profile (mm), *ρ_b_* is the bulk density of the soil (g cm^−^³), and *z*_tot_ is the depth from the soil surface to the bottom of the soil profile (mm).

The original model structure was modified by updating the surface temperature calculation to account for the effect of mulching, as shown in Equation 17:

(17) $$T_{sur}=scfT_{soil}\left(1,d_{n-1}\right)+\left(1-scf\right)T_{bare}$$

where *scf* is the surface cover factor, $T_{\text{soil}}\left(1,d_{n-1}\right)$ is the soil temperature of the first layer on the previous day (°C), and *T*_bare_ is the bare soil surface temperature (°C). This equation reflects the regulation of surface soil temperature by surface cover. The soil temperature from the previous day is weighted with the bare soil surface temperature using the factor *scf*, which accounts for the lag effect of the cover.

The bare soil surface temperature is calculated from daily average air temperature and a radiation term using Equations 18 and 19:

(18) $$T_{bare}={\bar{T}}_{av}+\epsilon_{sr}\frac{\left(T_{max}-T_{min}\right)}{2}$$

(19) $$\epsilon_{sr}=\frac{H_{day}\left(1-\alpha \right)-14}{20}$$

where *T*_bare_ is the surface temperature (°C) without mulching, *ϵ*_sr_ is the radiation term, *H*_day_ is the solar radiation reaching the soil surface (MJ m^−2^ d^−1^), and α is the surface albedo.

The surface cover factor *scf* and its canopy and residue components are calculated using Equations 20–22:

(20) $$scf=min\left(f_{upper},max\left(f_{cc},f_{rsd}\right)\right)$$

(21) $$f_{cc}=\frac{CC}{\left(CC+e^{\left(0.24-0.24\times CC\right)}\right)}$$

(22) $$f_{rsd}=\frac{RSD}{\left(RSD+e^{\left(0.12-0.12\times RSD\right)}\right)}$$

where *scf* is the weight factor for the effect of surface cover, which is 0.0 for bare soil and approaches 1.0 as the cover increases. *RSD* is the total amount of crop residue present on the day (t ha^−1^), and *f*_upper_ is a parameter that sets the upper limit for the vegetation cover coefficient.

Further details on the theoretical basis, parameter determination, and sensitivity analysis of the ST submodule are provided in **Supplementary Methods S1**.

#### 2.5.2 Crop growth and development under mulching

In this study, AquaCrop 7.0 was used to simulate maize growth and development based on GDD that describe the crop growth cycle. GDD is calculated using Equation 23 (Raes et al., 2009):

(23) $$GDD=\{\begin{array}{c} T_{u}-T_{b}\;,\;T_{ave}>T_{u}\\ \;T_{ave}-T_{b}\;,\;T_{b}\leq T_{ave}\leq T_{u}\\ 0\;,\;T_{ave}<T_{b} \end{array}$$

where *T*_ave_ is the daily average temperature (°C), and *T_u_* and *T_b_* are the upper and base temperature limits for crop growth, set at 30°C and 8°C, respectively.

AquaCrop divides the crop growth cycle into four growth stages and adjusts crop growth simulation according to different stages (**Table 3**). Research shows that the regulatory effect of surface mulch on soil temperature is most significant during maize emergence and seedling stages (Liang et al., 2017). As the canopy closes, the shading effect of vegetation itself increases, and the influence of mulch gradually weakens (Sun et al., 2019; Zhang et al., 2023). Therefore, surface mulch mainly affects soil temperature in stages 1 and 2 of AquaCrop. Surface temperature can significantly distinguish the differences between mulched and unmulched conditions, so this study uses surface soil temperature instead of air temperature in stages 1 and 2 to simulate crop growth and development under surface mulch conditions. The improved GDD calculation is presented in Equation 24:

**Table 3:** Calculation basis for GDD at each crop-growth stage.

Stage	Description	GDD selection
Stage 1	Emergence — 10% CC	Soil temperature
Stage 2	10% CC — Maximum CC	Soil temperature
Stage 3	Maximum CC — Beginning of senescence	Air temperature
Stage 4	Beginning of senescence — Maturity	Air temperature

(24) $$GDD=\{\begin{array}{c} T_{u}-T_{b}\;,\;T_{sur}>T_{u}\\ \;T_{sur}-T_{b}\;,\;T_{b}\leq T_{sur}\leq T_{u}\\ 0\;,\;T_{sur}<T_{b} \end{array}$$

### 2.6 Model parameter calibration and evaluation

In this study, the CK treatment dataset was used for baseline model calibration, whereas the datasets from the three straw-mulching treatments (CS, SS, and WS) were used to evaluate model performance under different mulching management practices (Chu et al., 2022). Differences among the mulching treatments were represented only through management inputs corresponding to the actual experimental treatments, including mulch coverage fraction, the mulch-induced soil evaporation adjustment factor, and the amount of surface residue. This parameterization strategy avoids artificially modifying crop cultivar parameters or soil properties among treatments, thereby ensuring that differences in model simulations primarily reflect the effects of the actual management practices.

The ST submodule uses daily solar radiation as an energy input to calculate soil surface temperature. Soil bulk density *ρ_b_* and soil albedo α were obtained from field measurements, whereas λ was calibrated against soil temperature observations from the CK treatment using a trial-and-error approach and was subsequently kept constant across all treatments. For the mulching treatments, the surface residue amount RSD was estimated from field conditions at the beginning of the growing season, accounting for straw-mulch losses over the winter period.

The calibration of AquaCrop parameters followed a stepwise iterative trial-and-error approach, in which relevant parameters were adjusted to minimize discrepancies between observed and simulated CC, soil water content, aboveground biomass, and grain yield (Sandhu and Irmak, 2019). Following the parameterization approach of Vanuytrecht et al. (2014), crop phenology was first calibrated by adjusting the GDD requirements for key developmental stages, including emergence, maximum canopy cover, flowering, onset of senescence, and maturity, until the simulated crop development approximated the field observations. Soil hydraulic parameters were then adjusted based on the observed soil water content. Subsequently, parameters controlling canopy expansion and decline, including the CGC and CDC, were adjusted to match the observed canopy dynamics. In addition, maize responses to water stress were calibrated by adjusting selected parameters related to canopy expansion and senescence. The WP* was then determined using the observed aboveground biomass and simulated cumulative crop transpiration. Finally, HI_0_ was determined using the observed grain yield and aboveground biomass at harvest.

The trial-and-error calibration approach may be influenced by researcher experience and parameter equifinality. To reduce these effects, calibration was limited to the key parameters requiring adjustment, while the remaining parameters were retained at their default or previously established values. The initial values, calibration ranges, and final calibrated values of the soil hydraulic parameters for each soil layer are presented in **Table 4**. The main parameters of the AquaCrop-ST model are summarized in **Table 5**.

**Table 4:** Calibration range and final values of soil hydraulic parameters.

Depth (cm)	*θ_FC_* (cm^3^ cm^−3^)				*θ_PWP_* (cm^3^ cm^−3^)			
	Initial	Min	Max	Final	Initial	Min	Max	Final
0–20	0.271	0.190	0.352	0.31	0.13	0.091	0.169	0.15
20–40	0.282	0.197	0.367	0.34	0.10	0.07	0.13	0.121
40–60	0.296	0.207	0.385	0.30	0.09	0.063	0.117	0.11
60–80	0.30	0.210	0.390	0.30	0.10	0.07	0.13	0.122

**Table 5:** AquaCrop-ST model main parameters.

Model	Parameter description	Parameter value	Unit	Source
AquaCrop	Base temperature	8	°C	Default
	Upper temperature	30	°C	Default
	Canopy growth coefficient (CGC)	0.012	GDD^−1^	Calibrated
	Canopy decline coefficient (CDC)	0.010	GDD^−1^	Calibrated
	Maximum canopy cover (CCx)	0.90	%	Calibrated
	Shape parameter for root growth pattern	1.3	–	Default
	Decline of crop coefficient	0.3	%	Default
	Normalized water productivity (WP*)	33	g m^−2^	Calibrated
	Maximum increase of HI	15	%	Default
	Upper limit for canopy expansion stress	0.15	%	Calibrated
	Lower limit for canopy expansion stress	0.70	%	Calibrated
	Shape parameter of canopy expansion under water stress	3	–	Calibrated
	Upper threshold for stomatal closure	0.69	%	Default
	Response curve shape for stomatal control	6	–	Default
	Upper threshold for early senescence	0.69	%	Default
	Response curve shape for senescence	6	–	Calibrated
	Upper threshold for pollination stress	0.8	%	Default
	Lower temperature limit for pollination	10	°C	Default
	Upper temperature limit for pollination	40	°C	Default
	GDD emergence	80	°C	Measured
	GDD senescence	1650	°C	Measured
	GDD maturity	1885	°C	Measured
	GDD flowering	850	°C	Measured
	GDD yield formation	880	°C	Measured
	Duration of flowering period	180	°C	Measured
	Max root depth	1.9	m	Measured
	Min root depth	0.3	m	Default
	Reference harvest index (HIo)	45	%	Measured
ST	Lag coefficient (λ)	0.7	–	Calibrated
	soil bulk density (*ρ_b_*)	1.5	g cm^−^³	Measured
	Soil albedo (*α*)	0.2	–	Measured
	Upper limit of coverage coefficient (*f*_upper_)	0.9	–	Default

Model evaluation was conducted by comparing simulated and observed results under the CS, SS, and WS straw-mulching treatments. The evaluation variables included soil temperature, soil water content, CC, aboveground biomass, and grain yield. Model performance was evaluated using four statistical indicators (Equations 25–28): coefficient of determination (R^2^), root mean square error (RMSE), Nash-Sutcliffe efficiency coefficient (NSE), and percentage bias (PBIAS). An R^2^ closer to 1 indicates better consistency between simulated and observed trends; RMSE closer to 0 indicates higher simulation accuracy. NSE values range from (-∞, 1], with NSE > 0.5 typically considered acceptable. The ideal value for PBIAS is 0, with positive values indicating underestimation and negative values indicating overestimation. Generally, |PBIAS|≤25% is considered acceptable (Heng et al., 2009; Hsiao et al., 2009).

(25) $$R^{2}={\left[\frac{\sum_{i=1}^{n}\left(O_{i}-\bar{O}\right)\left(S_{i}-\bar{S}\right)}{\sqrt{\sum_{i=1}^{n}{\left(O_{i}-\bar{O}\right)}^{2}\sum_{i=1}^{n}{\left(S_{i}-\bar{S}\right)}^{2}}}\right]}^{2}$$

(26) $$RMSE=\sqrt{\frac{1}{n}\sum_{i=1}^{n}{\left(S_{i}-O_{i}\right)}^{2}}$$

(27) $$NSE=1-\frac{\sum_{i=1}^{n}{\left(O_{i}-S_{\text{i}}\right)}^{2}}{\sum_{i=1}^{n}{\left(O_{\text{i}}-\bar{O}\right)}^{2}}$$

(28) $$PBIAS=\frac{\sum_{i=1}^{n}\left(O_{i}-S_{i}\right)}{\sum_{i=1}^{n}O_{i}}\times 100$$

where *O_i_* represents the actual observed value, *S_i_* is the model-simulated value; $\bar{O}$ and $\bar{S}$ are the respective means; *n* is the total number of samples.

## 3 Results

### 3.1 Effects of mulching measures on soil temperature dynamics

Mulching treatments generally reduced diurnal soil temperature variation and attenuated extreme temperature fluctuations (**Figure 4**). During high-temperature periods, the maximum soil temperature under the CK treatment reached 29.01 °C, whereas CS, SS, and WS reduced the maximum temperature by 1.77, 3.58, and 4.46 °C, respectively (**Figure 4a**). As shown in **Figure 4b**, the CK treatment exhibited a pronounced warming trend during the early growth stage because of the absence of surface cover. With canopy development, increased shading reduced the amount of solar radiation reaching the soil surface, resulting in a subsequent decline in soil temperature. In contrast, straw mulching provided a persistent temperature-moderating effect throughout the growing season by reducing heat transfer to the soil. As the crop canopy developed, the combined effects of canopy shading and straw cover further reduced soil warming. Consequently, all three mulching treatments maintained lower soil temperatures than CK during the observation period (**Figures 4c–e**). The average temperature reductions under CS, SS, and WS were 0.76 ± 0.96 °C, 0.87 ± 0.75 °C, and 1.31 ± 0.98 °C, respectively, with maximum reductions of 3.77 °C, 3.44 °C, and 4.32 °C, respectively.

**Figure 4:**
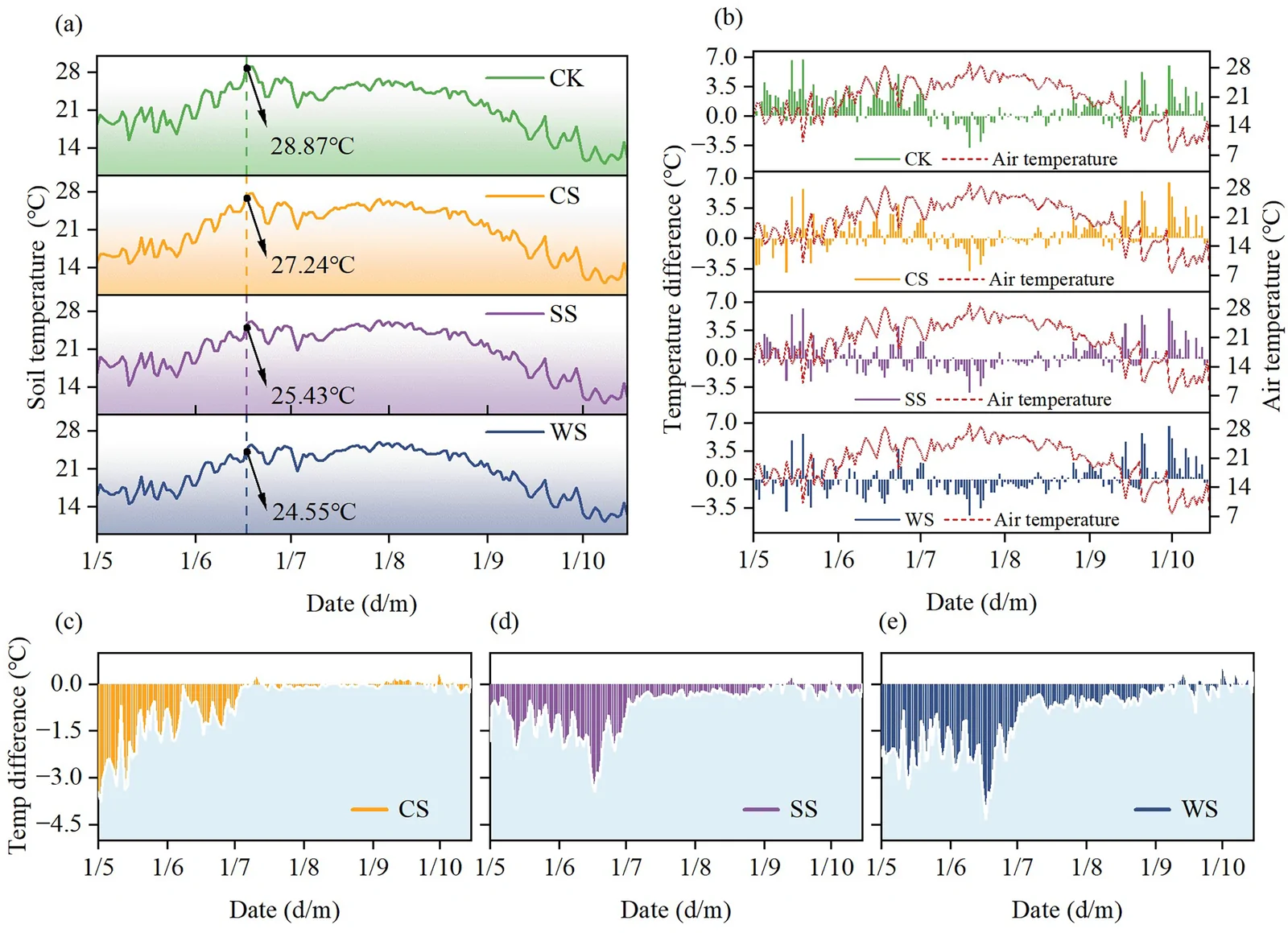
Effects of different mulch treatments on soil temperature dynamics. **(a)** Daily variations in soil temperature under different mulch treatments; **(b)** daily differences between soil and air temperatures under different mulch treatments; **(c–e)** soil temperature differences between each mulch treatment and CK.

Soil temperature patterns under mulching treatments showed significant differences among growth stages (**Figure 5**). During the sowing-to-emergence stage (**Figure 5a**), the temperature distribution peak of CK was concentrated at 18–20 °C, while the peaks of the mulching treatments shifted toward lower temperatures, with CS at 17–19 °C and SS and WS at 16–18 °C; the temperature distributions were also more dispersed. The mean soil temperature of CK was 19.1 °C, while those of CS, SS, and WS were 16.4, 18.0, and 16.8 °C, respectively, representing reductions of 1.1–2.7 °C relative to CK. Among the mulching treatments, CS showed the largest temperature reduction (**Figure 5e**). During the same period, soil accumulated temperature under the mulching treatments was 10–24% lower than that under CK (**Figure 5i**), indicating that mulching reduced early-stage soil warming and delayed crop emergence.

**Figure 5:**
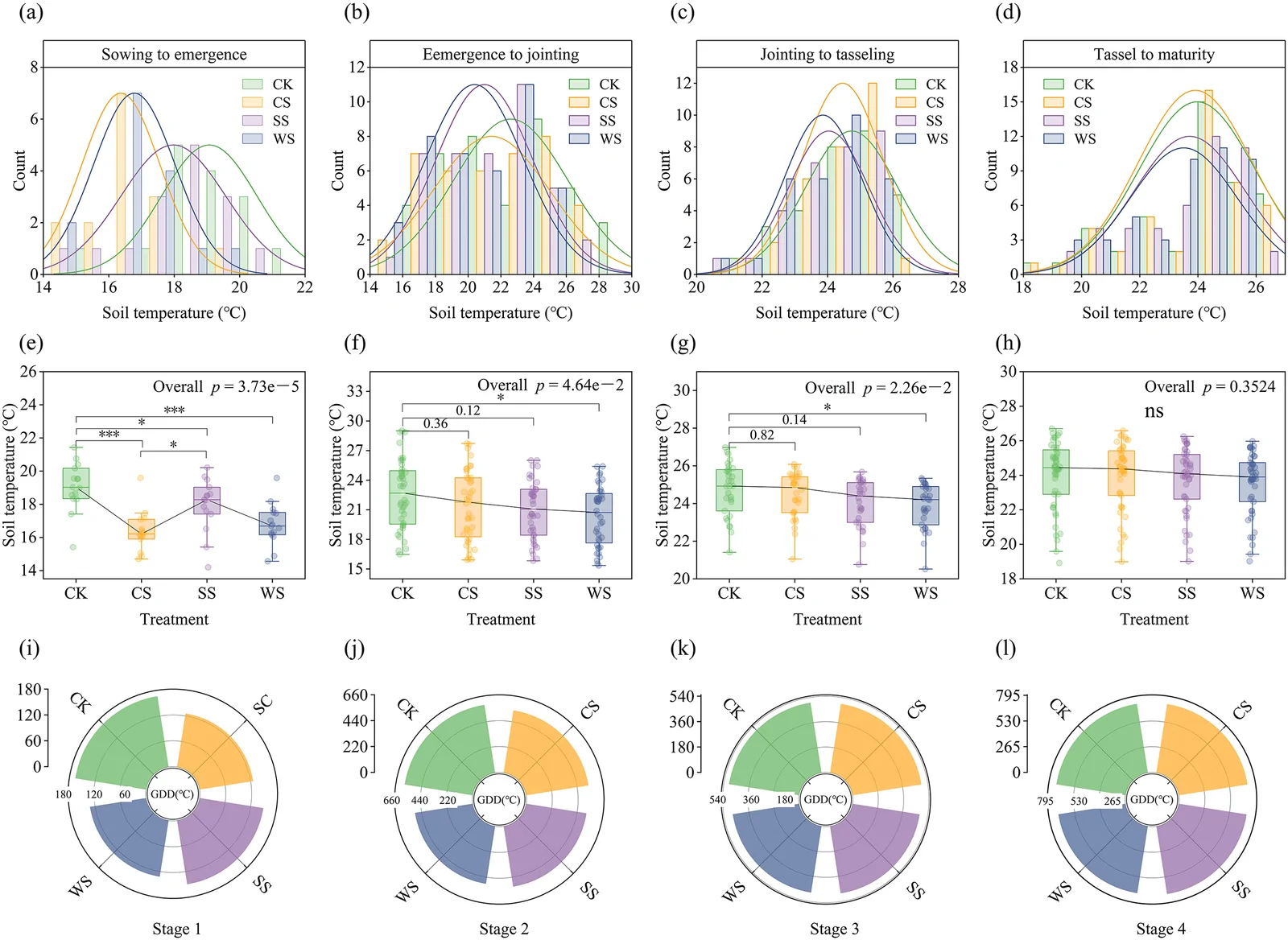
Soil temperature characteristics under different mulch treatments during maize growth stages. **(a–d)** Histograms and fitted curves of soil temperature distributions for each growth stage; **(e–h)** boxplots of soil temperature for each treatment and stage (*p< 0.05, **p< 0.01, ***p< 0.001; ns, not significant); **(i–l)** accumulated soil growing degree days (GDD) for each stage.

During the canopy expansion stage (**Figures 5b, c**), temperatures of all treatments increased overall, but the differences among treatments narrowed to 0.8–1.6 °C, with accumulated temperature remaining 6–13% lower under the mulching treatments than under CK. During this stage, the temperature-reduction effect of CS weakened, while WS maintained a relatively stable effect and showed better mitigation of high soil temperatures.

During the tasseling-to-maturity stage (**Figure 5d**), temperature peaks of all mulching treatments tended to converge with CK, and the influence of mulching on soil temperature in the late growth stage weakened substantially, with a temperature difference of less than 0.5 °C and no significant overall difference (p = 0.352, **Figure 5h**). Additionally, the temperature reduction under WS was significantly greater than those under CS and SS (p< 0.05), while the difference between CS and SS treatments was not significant (p > 0.05).

### 3.2 Soil temperature simulation performance

The simulation results of the newly added soil temperature module for the 10 cm soil layer are shown in **Figure 6**. Overall, the model accurately reproduced soil temperature variation patterns under different mulching treatments, with RMSE values of 0.93–1.06 °C, R^2^ values of 0.92–0.95, and NSE values of 0.88–0.94 (**Figures 6a–d**). During the seedling stage, temperature differences between treatments were large, while the model assumed a constant temperature-reduction effect throughout the entire growth cycle. Because the temperature-reduction parameter did not vary over time, the PBIAS values during the seedling stage ranged from 0.3–3.4%. In the late growth stage, the model generally overestimated the temperature-reduction effect of mulching, which was mainly attributed to gradual straw decomposition over time and the resulting weakening of the mulching effect. Overall, the newly added module was able to provide good model performance in simulating soil temperature under different surface mulching measures.

**Figure 6:**
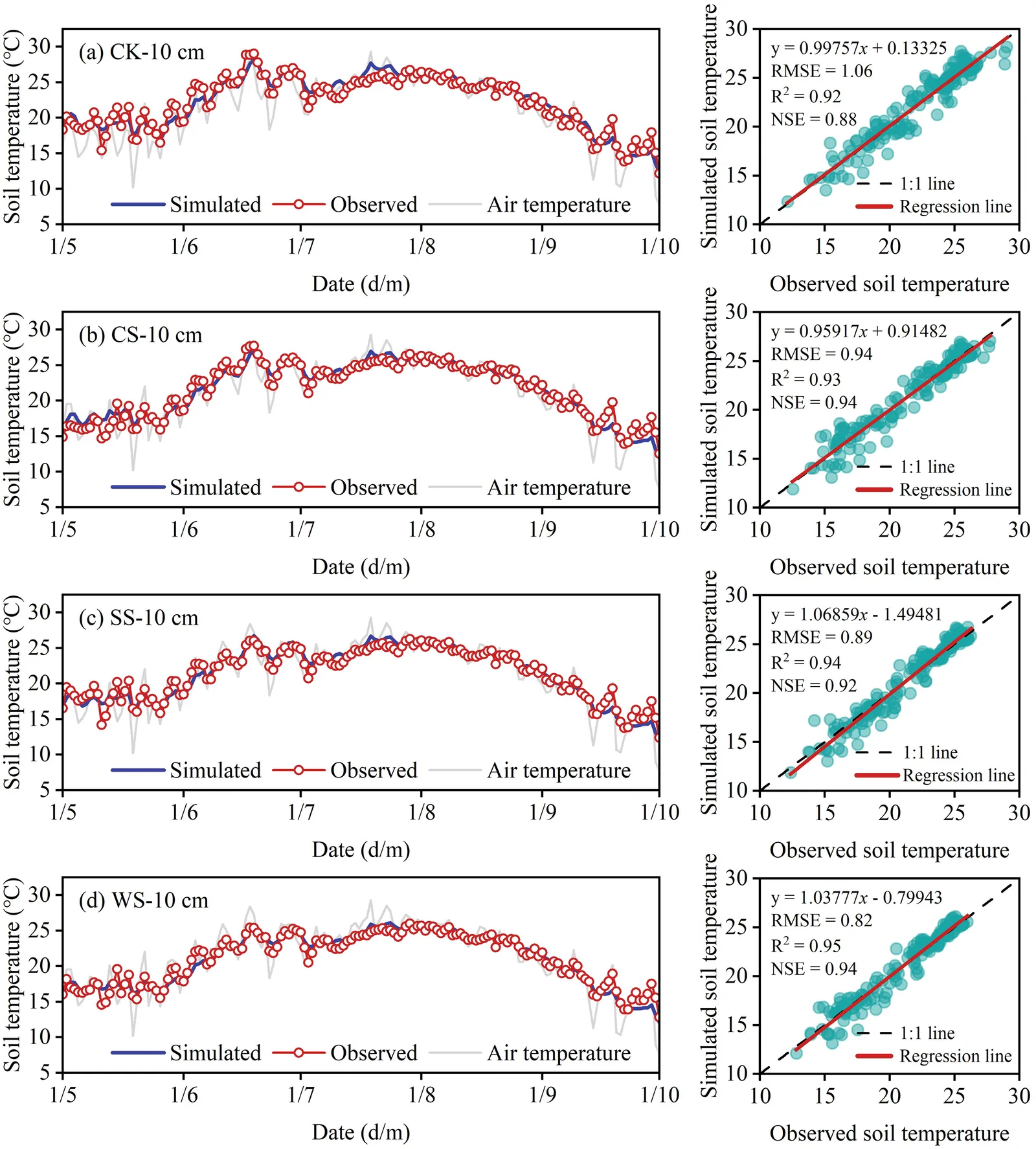
Simulated versus observed soil temperature at 10 cm depth under different mulch treatments: **(a)** CK, **(b)** CS, **(c)** SS, and **(d)** WS. The left panels show temporal variations, and the right panels compare simulated and observed values.

**Figure 7** shows the seasonal variation in soil profile temperature under different mulching treatments. Overall, the model reproduced the vertical and seasonal variation patterns of soil temperature well. The surface soil was more strongly affected by air temperature fluctuations and exhibited greater temperature variability, whereas soil temperature became progressively more stable with increasing depth. Under the mulching treatments (CS, SS, and WS), soil profile temperatures during high-temperature periods were generally lower than those under CK, and the magnitude of temperature reduction decreased with soil depth, indicating that straw mulching moderated heat transfer and soil temperature fluctuations. At 40–100 cm depth, the model showed overall stable performance. R^2^ = 0.76–0.97, NSE = 0.51–0.78, and PBIAS ranged within ±8% (**Table 6**), indicating that the model was able to reliably capture deep soil temperature variation trends. The 40 cm layer was strongly influenced by surface driving forces, with obvious diurnal temperature signals, and the model showed high simulation accuracy, with R^2^ > 0.93 and PBIAS< 6.1%. However, in deep layers (80–100 cm), temperature fluctuation amplitude was small, and, due to the influence of heat-conduction lag and vertical variation in soil thermal properties, NSE decreased to 0.55–0.58 with slightly increased bias. This indicates that the model performed well in simulating temperature in shallow and middle layers, while deep-layer simulation was more significantly limited by physical processes.

**Figure 7:**
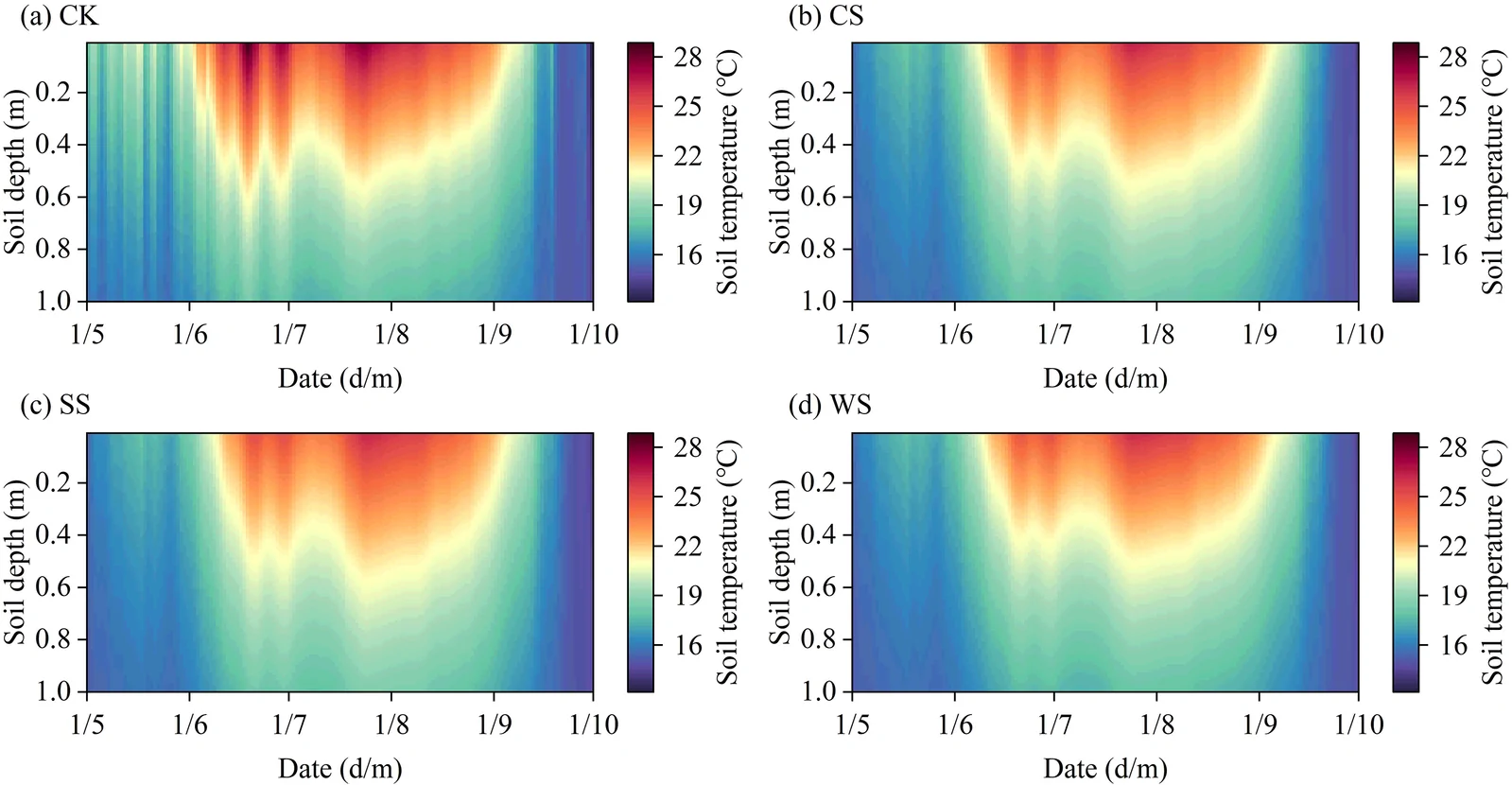
Heatmaps of simulated soil temperature profiles under different mulch treatments: **(a)** CK, **(b)** CS, **(c)** SS, and **(d)** WS.

**Table 6:** Statistical evaluation of model performance for simulated and observed soil temperatures at 40–100 cm depth.

Treatment	Depth (cm)	R^2^	NSE	PBIAS (%)
CK	40	0.93	0.78	6.10
	60	0.86	0.64	7.37
	80	0.88	0.55	8.45
	100	0.89	0.58	8.24
CS	40	0.96	0.78	1.09
	60	0.90	0.64	6.06
	80	0.81	0.72	5.24
	100	0.82	0.51	0.99
SS	40	0.97	0.78	6.02
	60	0.91	0.64	7.13
	80	0.94	0.61	5.42
	100	0.88	0.54	6.59
WS	40	0.95	0.76	-0.16
	60	0.94	0.63	3.27
	80	0.83	0.59	1.85
	100	0.76	0.66	4.16

### 3.3 Improvement in the simulation of maize growth and yield

Results showed that the original model generally underestimated crop growth progress under straw mulching conditions, while the improved model performed more accurately in CC simulation. Across all treatments, RMSE significantly decreased from 33.49–33.73% to 3.13–8.13%, with NSE > 0.93 (**Figures 8a–c**; **Table 7**). Meanwhile, the model was able to well reproduce the growth period delay effect under mulching treatments: compared to CK, CS, SS, and WS treatments extended the maize growing period by 8, 9, and 12 days, respectively. This delay was mainly attributed to the reduced surface soil accumulated temperature during the seedling stage, which slowed down the accumulation of GDD for maize meristem development. Notably, although soil temperature differences between CS and SS treatments were relatively small, the model was still able to capture a 1-day difference in growth period.

**Figure 8:**
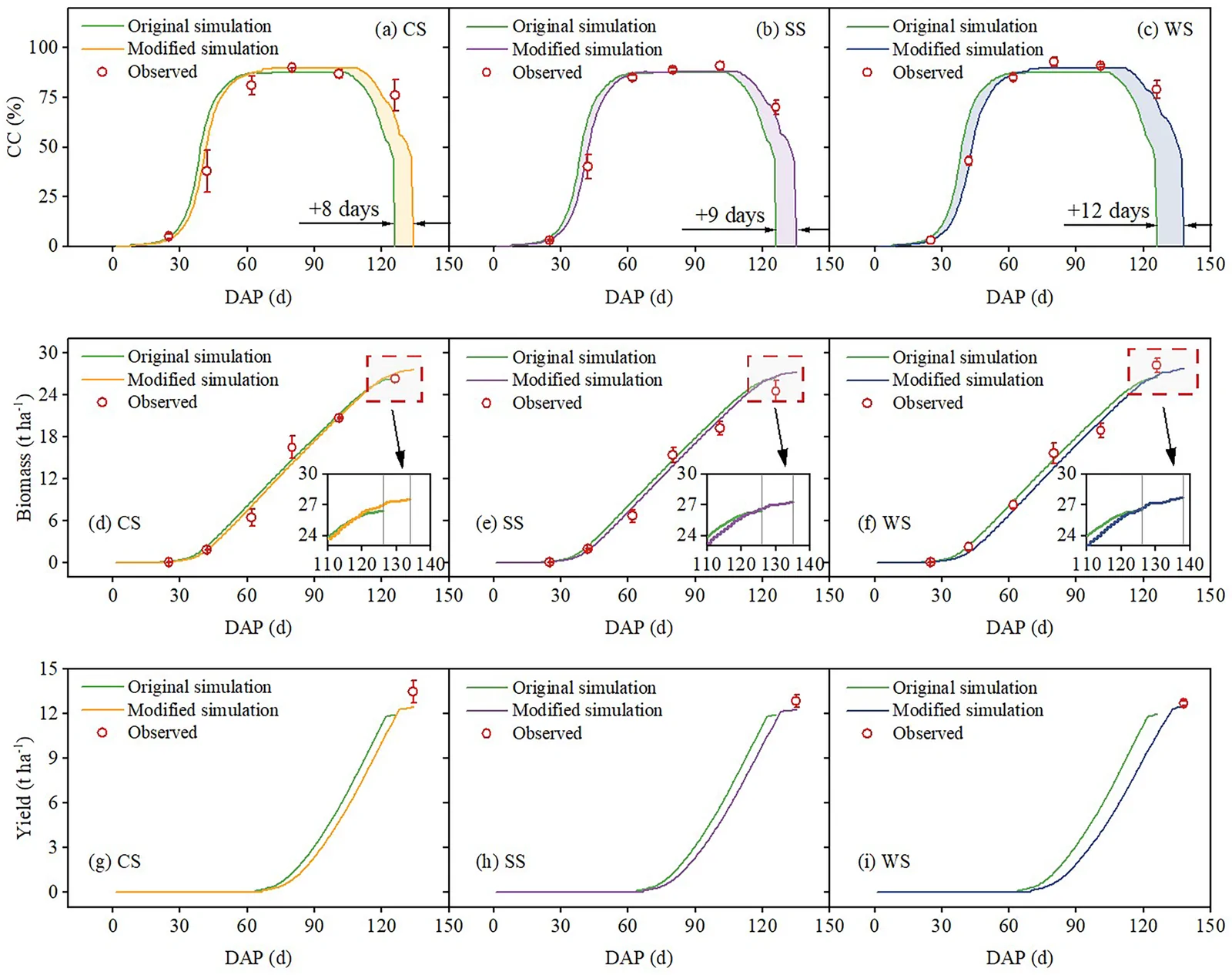
Model performance of original and modified simulations evaluated against observed data for canopy cover **(a–c)**, aboveground biomass **(d–f)**, and yield **(g–i)** under different treatments. DAP in the image stands for Days After Planting.

**Table 7:** Model evaluation results under different treatments before (Original) and after (Modified) improvement.

Parameter	Treatment	Original			Modified		
		RMSE	NSE	PBIAS(%)	RMSE	NSE	PBIAS(%)
CC	CS	33.49	-0.16	-10.56	8.13	0.93	3.16
	SS	30.84	0.07	-10.79	4.36	0.98	0.5
	WS	33.73	-0.07	-15.03	3.13	0.99	-2.69
Biomass	CS	1.2	0.98	6.37	1.03	0.99	2.37
	SS	1.28	0.98	9.14	0.86	0.99	3.04
	WS	1.07	0.99	3.42	0.82	0.99	-2.88
Yield	CS	1.46	–	-10.79	1.09	–	-8.06
	SS	0.83	–	-6.43	0.59	–	-4.58
	WS	0.72	–	-5.7	0.2	–	-1.59

This growth period change reflects the direct influence of soil hydrothermal environment on crop growth, development, and physiological activities through multiple pathways. This study found that mulching treatments have comprehensive effects on crop growth by regulating soil temperature and water availability. During emergence and early growth stages, mulching treatments generally reduced soil temperature and delayed accumulated temperature accumulation (**Figures 4**, **5**), thereby causing delayed emergence time. This effect was most pronounced in WS treatment, where lower soil temperature may have inhibited early growth rate to some extent. However, this early growth “disadvantage” gradually transformed into mid-to-late stage advantages as the growth process progressed. Mulching treatments provided more stable growth environments for crops by mitigating extreme temperature fluctuations and improving soil water conditions.

The delayed growth period provided crops with longer biomass accumulation time. Although the original model could better reflect biomass accumulation trends, the original model showed some deviation in later stages (**Figures 8d–f**). After improvement, RMSE decreased from 1.07–1.28 t ha^−1^ to 0.82–1.03 t ha^−1^, and PBIAS decreased across all treatments (e.g., SS decreased from 9.14% to 3.04%), with improved biomass simulation accuracy.

The delay in growth period not only affects dry matter accumulation processes but also influences harvest index formation. The improved model showed certain yield increase trends, with CS, SS, and WS treatment yields increasing by 4.23%, 3.13%, and 4.58%, respectively, compared to original simulations, and RMSE reduction reaching 25–72%. This yield increase was mainly attributed to extended growth periods, providing crops with longer photosynthesis and dry matter accumulation time. However, the relationship between growth period delay and yield increase is not linearly positive: excessively long growth periods may expose crops to more stress factors, thereby limiting yield potential.

Grain yield tended to increase under straw mulching treatments. The measured grain yields were 13.50, 12.87, 12.70, and 12.30 t ha^−1^ for CS, SS, WS, and CK, respectively. Compared with the non-mulched CK, grain yield increased by 9.76%, 4.63%, and 3.25% under CS, SS, and WS, respectively. The greater temperature reduction under WS did not result in the highest yield, suggesting that excessive cooling during the early growth stage may have limited crop establishment. Grain yield under CS was significantly higher than that under SS and WS, which may be related to differences in mulch structure, water retention, air permeability, and decomposition characteristics. These differences likely contributed to improved soil hydrothermal conditions and more favorable conditions for crop growth.

### 3.4 Effects of straw mulching on soil water content

Soil water content at 20 cm layer was able to significantly reflect differences between mulching and non-mulching treatments. **Figure 9** shows the simulation results of improved model for 20 cm soil water content under different treatments. Throughout the growing season, soil water content of CK, CS, SS, and WS treatments were 27.75 ± 4.38, 36.90 ± 4.36, 33.07 ± 5.07, and 34.22 ± 6.58%, respectively, with relative increase rates of mulching treatments compared to CK being CS 33.0%, SS 19.2%, and WS 23.3%. These results indicate that CS and WS had significant effects on improving surface soil water content.

**Figure 9:**
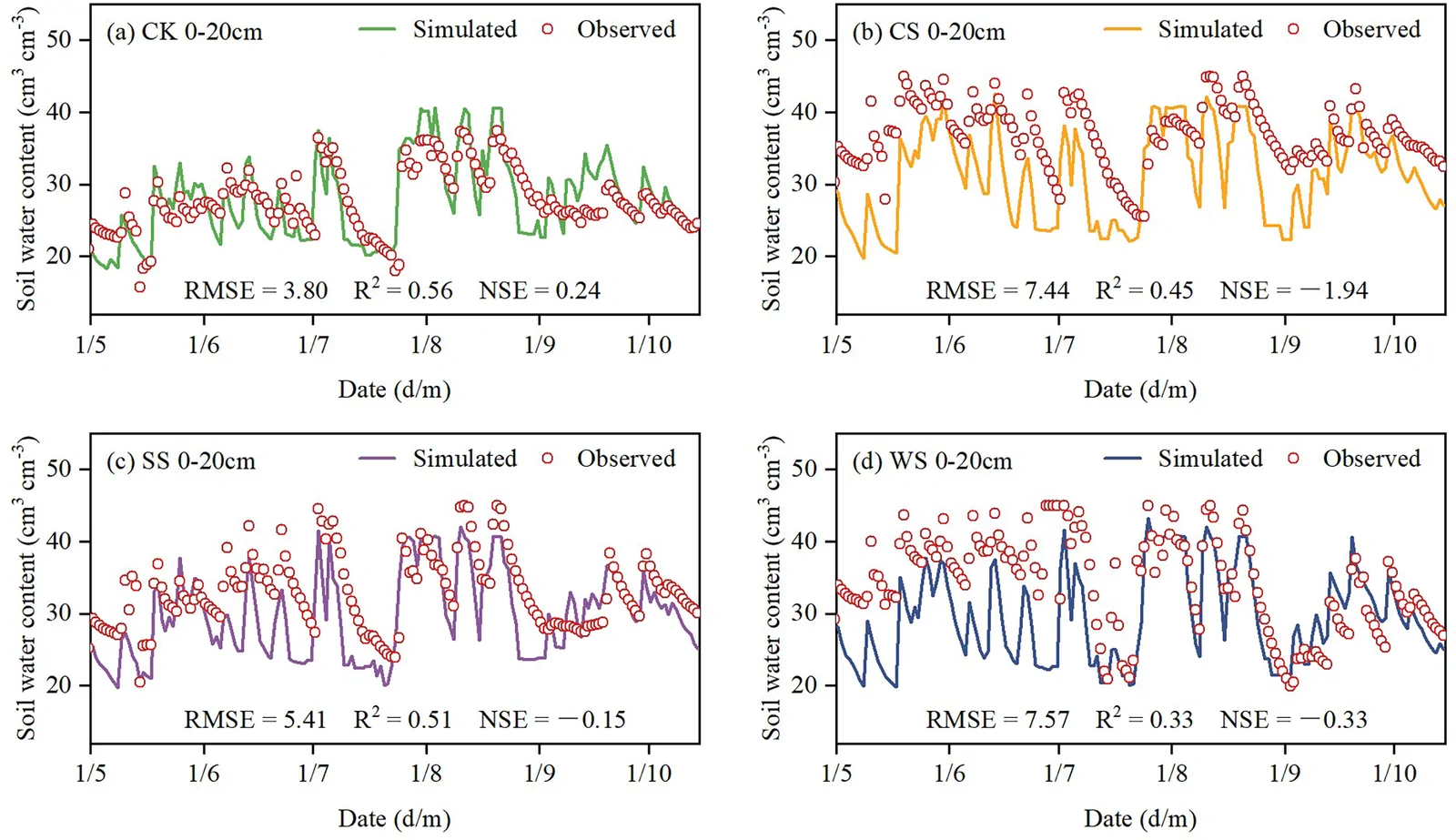
Simulated and observed soil water content (0–20 cm) under different mulching treatments: **(a)** CK, **(b)** CS, **(c)** SS, and **(d)** WS.

However, soil water content simulation under mulching treatments was generally underestimated. CK treatment simulation results were relatively accurate, with RMSE of 3.80, R^2^ of 0.56, and NSE of 0.24 (**Figure 9a**), indicating that the model performed well overall in predicting surface soil water content under non-mulching conditions. In contrast, mulching treatments (CS, SS, WS) had RMSE of 7.44, 5.41, and 7.57, respectively, R^2^ of 0.45, 0.51, and 0.33, respectively, and NSE of -1.94, -0.15, and -0.33 (**Figures 9b–d**), respectively, showing significantly declined model performance for surface soil water content under mulching conditions. In particular, the NSE values for all three mulching treatments were negative, which generally underestimated the water-retention effect of straw mulching.

**Figure 10** shows the evaporation simulation effects of the improved model under different treatments. The delayed growth period increased transpiration days for mulching treatments, but transpiration differences among treatments were small, indicating that surface soil water content was not a limiting factor for transpiration. CK treatment showed higher evaporation throughout the growth cycle, with differences most pronounced during early crop growth stages. Straw mulching treatments (CS, SS, WS) generally reduced evaporation, with reductions of 25.6%, 30.9%, and 10.9%, respectively, compared to CK. Evaporation suppression effects of mulching treatments followed the order SS > CS > WS, with SS treatment showing the best evaporation suppression effect. This is because the effectiveness of AquaCrop mulching measures depends on soil water status and evaporation stage. WS and CS had higher initial water content; when water was sufficient, mulching measures mainly affected potential evaporation but actual evaporation remained high; when water was insufficient, mulching measures worked together with soil water conduction limitations to significantly reduce actual evaporation.

**Figure 10:**
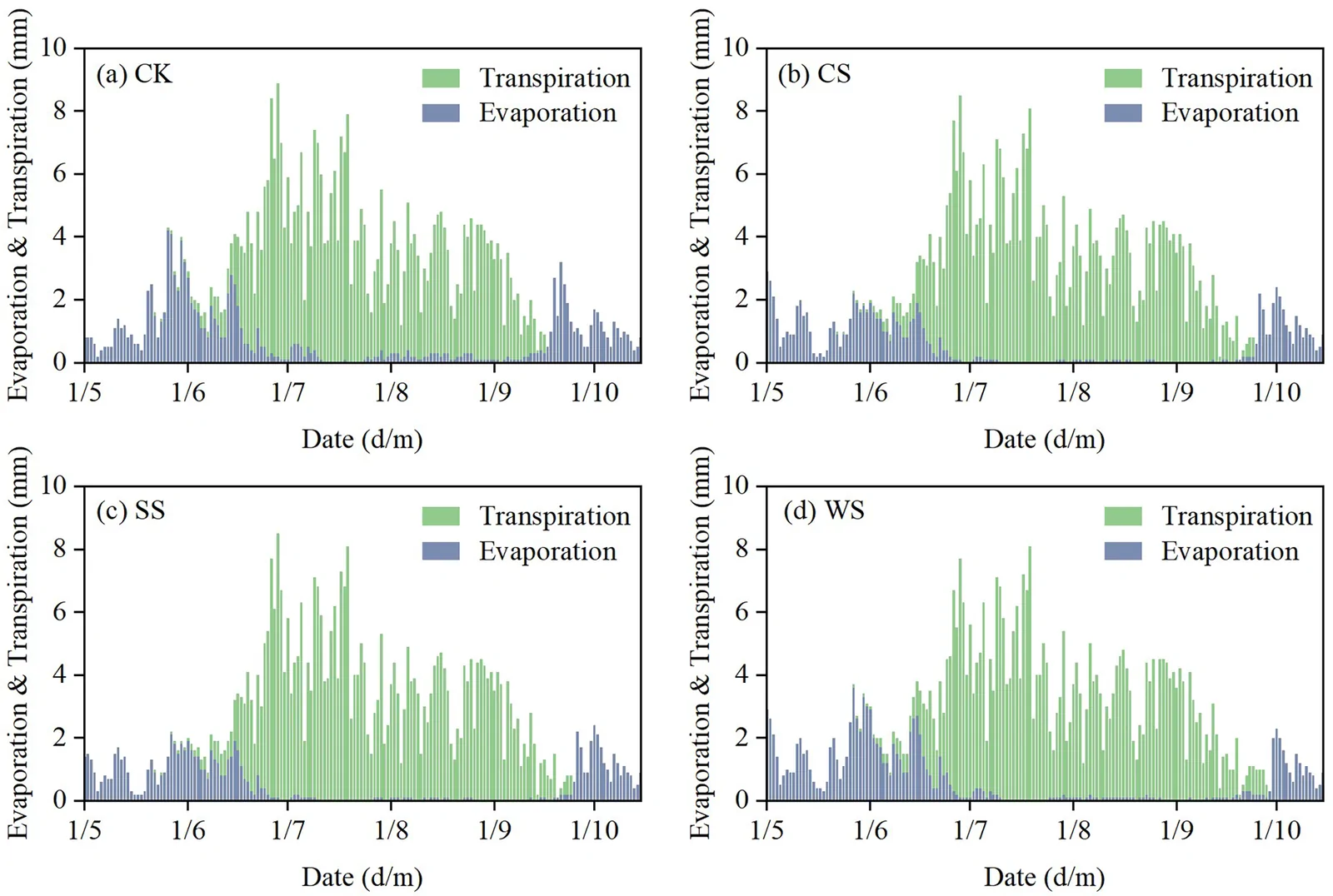
Daily transpiration and evaporation under different mulching treatments: **(a)** CK, **(b)** CS, **(c)** SS, and **(d)** WS.

**Figure 11** shows the simulated and measured soil water storage changes by AquaCrop. Overall, the model was able to reflect seasonal variation patterns of soil water: during periods of abundant precipitation or soil moisture, soil water storage of all treatments maintained at high levels with relatively small differences among treatments; while during drought stress in mid-to-late growing season, mulching treatments generally showed stronger water retention effects, with soil water storage significantly higher than CK, reflecting the role of mulching in reducing evaporation and improving soil water conditions.

**Figure 11:**
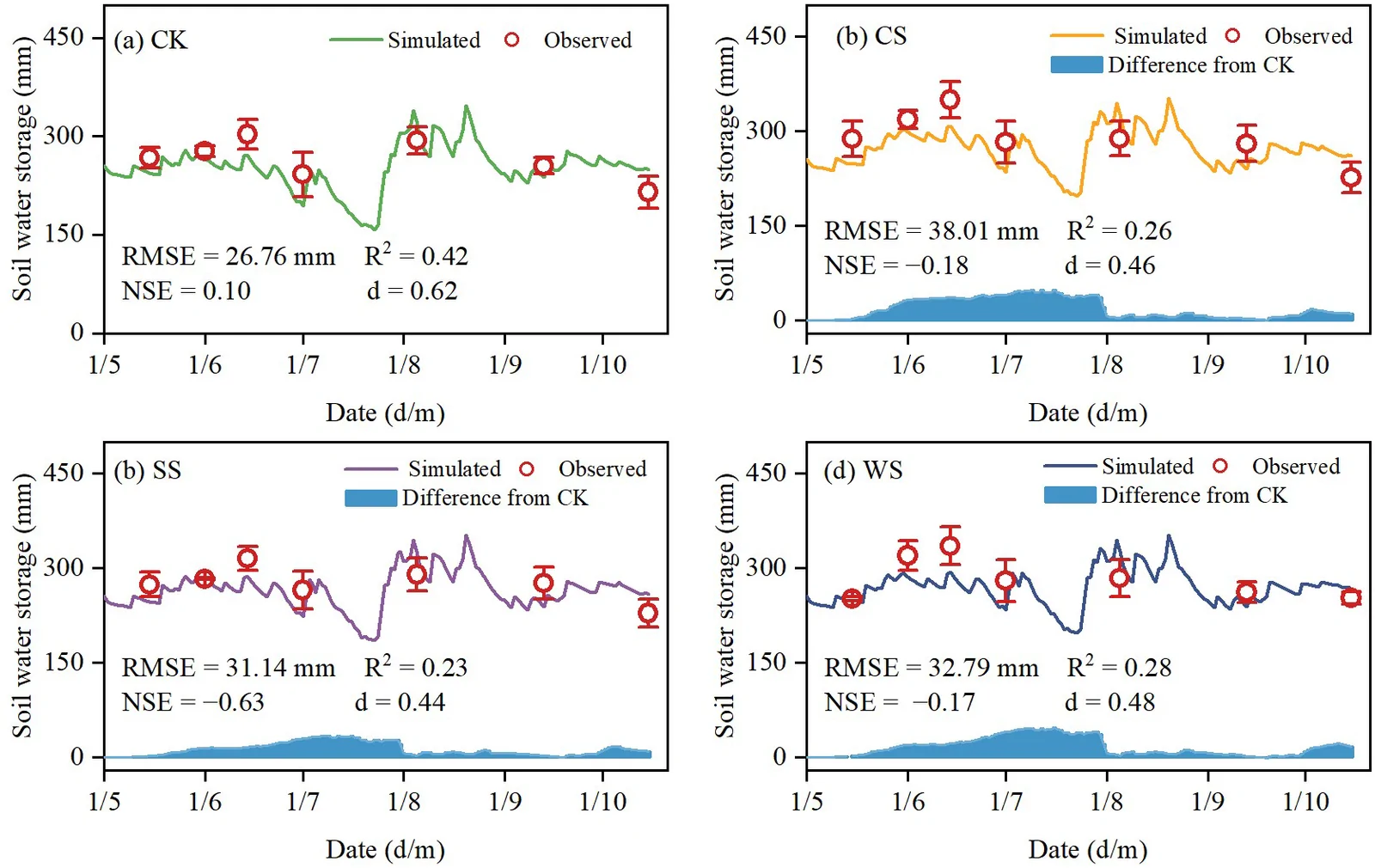
Simulated and observed soil water storage (0–100 cm) under different mulching treatments: **(a)** CK, **(b)** CS, **(c)** SS, and **(d)** WS. Blue shading in panels **(b–d)** indicates the difference from CK.

Mulching treatments all increased soil water storage, but to varying degrees. Compared to CK, CS, SS, and WS treatments showed relative increases of 9.8%, 4.4%, and 7.3%, respectively, with overall performance following the order CS > WS > SS > CK. During drought periods, these differences became more pronounced, with mulching treatments significantly slowing soil water decline rates and providing more stable water supply for crops. Mulching measures significantly increased available soil water during critical water-demanding periods such as jointing to grain filling, delaying the onset of water deficit, which was beneficial for maintaining canopy growth and thus provided guarantee for later biomass accumulation and yield formation.

For the CK treatment, the model yielded an RMSE of 26.76 mm, R^2^ of 0.42, and NSE of 0.10 (**Figure 11a**), indicating limited performance in reproducing soil water dynamics. Under mulching conditions, model performance declined further, particularly for the SS treatment, with an NSE of −0.63, which may be related to uneven spatial distribution of soil water under strip mulching and the AquaCrop model’s internal mechanisms not fully considering the effects of mulching measures on soil water transport. Nevertheless, CS and WS still reasonably reproduced seasonal fluctuations of soil water in overall trends.

## 4 Discussion

### 4.1 Effects of straw mulching on soil hydrothermal environment

Field measurements showed that straw mulching significantly improved the soil hydrothermal environment by altering surface energy exchange and water cycling. Overall, mulching treatments exhibited a dual regulatory effect of “cooling during high-temperature periods and insulating during low-temperature periods,” with lasting effects on reducing soil evaporation, increasing water storage, and buffering extreme temperatures (Chen et al., 2007; Liu et al., 2022).

In terms of temperature regulation, significant differences were observed among the different mulching methods. WS formed a thicker and more continuous mulch layer, which reduced surface soil temperature by 4.46 °C during high-temperature periods and maintained a relatively stable soil temperature throughout the growing season. CS showed the strongest cooling effect during the seedling-to-early-growth stage, but as the straw decomposed, its temperature-moderating effect gradually weakened. SS exhibited moderate temperature regulation but still played a significant role in buffering diurnal temperature fluctuations and extreme temperatures (Ma et al., 2024). It should be noted that excessive cooling during the seedling stage may delay meristematic development and reduce effective thermal accumulation, thereby delaying emergence and early growth.

In terms of water regulation, straw mulching generally increased surface soil moisture and reduced evaporation. The CS treatment showed the most significant increase in soil moisture in the 0–20 cm layer, with soil moisture increasing by 33.0% compared with CK. This was mainly due to the uniform distribution and good permeability of the mulch, which significantly inhibited evaporation and facilitated rainfall infiltration (Zhang et al., 2016). WS showed a 23.3% increase, but the overly thick mulch layer may hinder vertical water movement to some extent. SS showed a 19.2% increase, and although the effect was relatively limited, it significantly improved soil moisture supply during drought stress periods. Overall, mulching enhanced water availability during key crop growth periods through three pathways: reducing nonproductive evaporation, promoting water infiltration, and maintaining soil moisture storage. These effects helped stabilize leaf area and stomatal conductance (Zhang et al., 2015, 2022; Ma et al., 2024).

### 4.2 Effects of straw mulching on crop growth and yield

The improvements in the hydrothermal environment were ultimately reflected in maize phenology and yield formation. During the seedling stage, mulching treatments generally reduced the average soil temperature by 1.1–2.7 °C and soil accumulated temperature by 10–24%, resulting in delayed emergence, with WS showing the largest temperature reduction. As the growing season progressed, the effect of mulching gradually shifted from delaying early-stage development to promoting later-stage growth. By alleviating heat stress and water deficiency, mulching extended the growing period by 8–12 d, providing more time for photosynthetic assimilation and dry matter accumulation.

Regarding yield formation, CS achieved the highest increase (9.76%), demonstrating the dominant role of improved soil water availability in yield enhancement (Li et al., 2022; Liao et al., 2025). SS achieved a 4.63% yield increase through moderate adjustments to water and temperature, whereas WS, although more effective in mitigating high soil temperatures, showed relatively limited yield improvement (3.25%) because excessive cooling during the seedling stage partially restricted canopy establishment.

Although improper mulching management, such as applying an overly thick mulch layer under cold conditions or during early planting, may increase the risk of yield reduction by delaying emergence (Chen et al., 2007; Khaledian et al., 2012), numerous empirical studies have shown that appropriately designed straw mulching can generally improve soil water availability and the soil microbial environment, ultimately increasing crop yield (Liu et al., 2024; Zhang et al., 2024b, Zhang et al., 2024a). Therefore, the design of mulching practices should comprehensively consider regional climate, soil texture, and cultivar characteristics. The mulching method and intensity should be optimized to maintain an appropriate early-season soil temperature while ensuring stable water retention during the later growth stages, thereby achieving coordinated regulation of soil hydrothermal processes and stable yield improvement.

### 4.3 Comparison of improved model performance and limitations

In the original AquaCrop Field Management–Mulching module, the effect of mulching is represented primarily through a reduction in soil evaporation. Users specify the mulch type and surface coverage, and the model then reduces bare-soil evaporation according to an empirical relationship. The energy distribution mainly relied on CC, with evaporation being passively limited as the canopy closed. However, this approach did not explicitly consider the soil temperature profile, nor did it directly integrate soil temperature into phenology or GDD calculations, making it difficult to capture the direct effects of straw mulching on the thermal environment and crop growth processes. The results of this study further highlighted this limitation. The canopy-growth trajectories simulated by the original model under the mulching treatments were highly similar to those under the non-mulched condition, indicating that the existing mulch-management settings alone had only a limited influence on canopy development. Consequently, the original model generally failed to reproduce the observed mulch-induced effects on phenology-related crop growth and development, which subsequently resulted in biases in biomass and yield simulations. Previous studies have reached similar conclusions, indicating uncertainty in the applicability of AquaCrop under straw or plastic film mulching (Cheng et al., 2022).

Related studies have shown that surface soil temperature better reflects the differences between covered and uncovered treatments, with early growth stages often driven by shallow soil temperature rather than air temperature (Han et al., 2014; Hou et al., 2014; Zhao and Xiao, 2020; Zhao et al., 2023b). As a result, some studies have attempted to introduce soil temperature indicators into crop models to replace air temperature in order to improve simulations of emergence and early growth. However, the air temperature–soil temperature compensation method still shows deviations during planting or extreme climatic conditions (Shen et al., 2021; Zhang et al., 2023). Compared with these methods, this study improves upon the model by incorporating a soil temperature submodule (AquaCrop-ST), replacing air temperature with surface soil temperature for early accumulated temperature calculations, thereby explicitly reflecting the thermal environment regulation effect of straw mulching.

Simulation results showed that the improved model better reproduced the cooling of soil and slowing of accumulated temperature under mulching conditions, thus delaying emergence and early canopy expansion, and improving the accuracy of phenology and yield simulations. In CC and phenology simulations, NSE improved to 0.93–0.99, and RMSE decreased to 3–8%, allowing the model to reproduce the 8–12 day extension of the growing period caused by straw mulching (**Figures 8a–i**). In yield predictions, RMSE decreased by 25–72% compared to the original model, and the model was able to better distinguish yield differences between different mulching treatments.

The improved performance should also be considered in relation to the potential increase in model flexibility. To reduce the risks of parameter equifinality and overfitting, the ST module was deliberately kept parsimonious rather than introducing a large number of additional parameters related to multilayer heat-transfer properties, interfacial heat exchange, or soil thermal properties. The number of calibrated parameters was therefore restricted, while measured values and established default parameters were used wherever possible to avoid excessive freedom in parameter adjustment. Moreover, the improvement was observed across soil temperature, phenological development, canopy cover, biomass, and grain yield rather than only in the final yield. This process-level consistency suggests that the improved performance was associated with the explicit representation of soil thermal processes rather than simply increased fitting flexibility. Nevertheless, further validation across different years and environmental conditions is still needed to more rigorously assess parameter transferability and model robustness.

### 4.4 A potential simplification method

For practical use cases without continuous soil-temperature observations, further research could develop simplified modeling approaches. One possible strategy is to introduce a dynamic correction to daily effective accumulated temperature to represent the stronger thermal regulation of straw mulching during the early growth stage and its gradual weakening as canopy development and surface conditions change. Such an approach may provide a more practical alternative when detailed process-based simulation is not required, but the parameters of this empirical model remain to be further evaluated. In this study, this approach was preliminarily explored, with further details provided in the **Supplementary Material**.

### 4.5 Future research

Future studies should further evaluate the robustness and applicability of AquaCrop-ST using datasets from different years and locations. The representation of soil water processes under straw mulching should also be improved, particularly by accounting for the effects of straw mulch on rainfall interception, soil infiltration, surface runoff, and soil evaporation. In addition, the dynamic decomposition of straw throughout the crop growing season and the associated changes in surface coverage and hydrothermal regulation should be considered. These improvements would further enhance the model’s ability to simulate soil hydrothermal dynamics and crop growth responses under straw-mulching conditions.

## 5 Conclusion

1. Field experiments showed that straw mulching buffered soil temperature fluctuations, reduced early-season soil thermal accumulation, and improved soil water conditions, thereby affecting maize phenological development and yield formation. Clear differences were observed among mulching treatments: CS showed relative advantages in improving surface soil water conditions and maintaining higher yield, WS provided stronger and more persistent cooling and was associated with a more pronounced phenological delay, whereas SS exhibited an intermediate overall hydrothermal regulation effect. The yield responses among treatments reflected the integrated effects of mulching on soil hydrothermal conditions and crop growth rather than the influence of a single hydrothermal factor.
2. Compared with the original AquaCrop framework, AquaCrop-ST incorporates soil temperature processes into the existing water-response framework, thereby linking mulch-induced changes in the soil thermal environment with crop growth and development. This modification addresses the lack of an independent soil temperature module in the original model and improves the representation of canopy development, biomass accumulation, and yield formation under mulching conditions.

Overall, AquaCrop-ST provides a useful modeling framework for evaluating and optimizing straw-mulching management. Further validation across different years and environmental conditions will help assess its robustness and range of applicability and provide a basis for its application under future climate scenarios.

## Data Availability

The original contributions presented in the study are included in the article/**Supplementary Material**. Further inquiries can be directed to the corresponding author.
