# Supplementary material for: Integrating a soil temperature module into AquaCrop improves simulation of crop growth and yield under straw mulching
Source: Front Plant Sci. 2026 Oct 28;17:1968615. doi: 10.3389/fpls.2026.1968615 (PMC13752085; doi:10.3389/fpls.2026.1968615)
Supplement: Supplementary file 1 [file DataSheet1.pdf]

Supplementary Material

1 Supplementary Methods for the ST model

1.1 Theoretical basis of the ST submodule

The ST submodule used in this study does not explicitly solve the partial differential equation for soil heat conduction. Instead, it employs a daily recursive approach to calculate soil temperature at different depths based on the previous-day soil temperature, the current surface thermal condition, and the attenuation of temperature fluctuations with soil depth. The core relationship is expressed as:

$$T_{\text{soil}}(z, d_n) = \lambda T_{\text{soil}}(z, d_{n-1}) + (1-\lambda)\left[df\left(\overline{T}_{\text{Aair}} - T_{\text{sur}}\right) + T_{\text{sur}}\right] \quad \text{(S1)}$$

where $T_{\text{soil}}(z, d_n)$ and $T_{\text{soil}}(z, d_{n-1})$ represent soil temperature at depth z on the current and previous days, respectively; λ is the lag coefficient controlling the contribution of the previous-day soil thermal state to the current soil temperature; df is the depth factor describing the attenuation of surface temperature fluctuations with soil depth; and T_{sur} is the soil surface temperature.

Eqs. (11)–(22) in the main manuscript collectively describe three interconnected processes: temporal lag, vertical temperature damping, and regulation of the surface thermal condition by surface cover.

Eqs. (11)–(16) mainly describe the temporal lag and vertical damping of soil temperature. The damping depth (dd) is dynamically determined by soil bulk density, soil water conditions, and related soil properties, and subsequently affects the dimensionless depth and the depth factor (df). Thus, dd is not an independently prescribed correction parameter, but a dynamic variable controlling the attenuation of surface temperature fluctuations as they propagate into deeper soil layers.

Eqs. (17)–(22) mainly describe the surface thermal condition and the effect of surface cover. The surface temperature is expressed as:

$$T_{\text{sur}} = scf\, T_{\text{soil}}(1, d_{n-1}) + (1 - scf)T_{\text{bare}} \quad \text{(S2)}$$

where T_{bare} is the bare-soil surface temperature and scf is the surface cover factor. The value of scf controls the relative contributions of the previous-day upper-layer soil temperature and the current bare-soil surface temperature to the calculated surface temperature. Therefore, scf does not impose a fixed cooling effect; instead, it modifies the surface thermal condition that subsequently affects soil-temperature calculations.

The influence of scf on surface temperature can be further expressed as:

$$\frac{\partial T_{\text{sur}}}{\partial scf} = T_{\text{soil}}(1, d_{n-1}) - T_{\text{bare}} \quad \text{(S3)}$$

This relationship indicates that the effect of surface cover depends on the thermal contrast between the previous-day upper-layer soil temperature and the current bare-soil surface temperature. A larger temperature difference results in a stronger influence of surface cover on the calculated surface temperature.

Overall, surface cover regulates the surface thermal condition, the depth factor and damping depth determine the attenuation of temperature fluctuations through the soil profile, and the lag coefficient determines the persistence of the previous soil thermal state over time. Together, these processes determine the daily dynamics of soil temperature at different depths.

1.2 Determination of λ, dd, and scf

The three key quantities λ, dd, and scf play different roles in the ST submodule and are not parameters of the same type requiring simultaneous calibration.

The lag coefficient λ is a model parameter that must be determined before model application. It controls the contribution of the previous-day soil thermal state to the current-day soil temperature. In this study, λ was calibrated using the observed soil-temperature data from the CK treatment and was subsequently kept unchanged for all straw-mulching treatments. Therefore, the CS, SS, and WS treatments were not assigned treatment-specific values of λ.

In contrast, the damping depth dd is not an independently calibrated fixed parameter. It is dynamically calculated from soil bulk density, soil water content, and related soil properties. Variations in dd subsequently affect the depth factor df, thereby regulating the attenuation of surface temperature fluctuations with soil depth. Consequently, dd varies dynamically in response to changes in soil conditions.

Similarly, scf is not a fixed parameter calibrated separately for different straw-mulching treatments. In the modified ST submodule, scf is dynamically calculated according to canopy cover and surface straw residue conditions. Therefore, the effects of straw mulching on soil temperature are not represented by assigning treatment-specific empirical temperature-correction coefficients. Instead, differences in surface cover modify the calculated surface thermal condition, which subsequently affects soil temperature.

1.3 Comparison of the original and ST model

The modified ST submodule retains the basic daily recursive framework of the SWAT soil-temperature formulation on which it was based, including temporal lag, depth-dependent damping, and the effects of meteorological and soil conditions on soil temperature. The principal modification concerns the representation of surface cover and its dynamic coupling with canopy development in AquaCrop. A detailed comparison is provided in Table S1.

Table S1. Comparison between the original soil-temperature formulation and the modified ST submodule

Component	Original soil-temperature calculation method	Modified ST submodule
Temporal resolution	Daily	Daily

Component	Original soil-temperature calculation method	Modified ST submodule
Previous-day soil temperature	Considered	Retained
Temporal lag effect	Considered	Retained
Depth-dependent damping	Calculated according to soil properties and water conditions	Retained
Bare-soil surface temperature	Calculated from air temperature, solar radiation, albedo, etc.	Retained
Surface cover effect	The comprehensive impact of vegetation biomass and surface residues	Canopy cover and surface straw residue are considered separately
Canopy representation	Not included	Coupled with canopy cover (CC) in AquaCrop
Coupling with AquaCrop crop growth	Not included	Dynamically coupled with AquaCrop crop growth

The purpose of the present modification was therefore not to establish a completely different soil-temperature model. Rather, the original daily recursive and depth-damping framework was retained, while the representation of surface thermal conditions under straw mulching was adapted to the AquaCrop framework. This modification allows canopy development, surface straw residue, and soil temperature to be dynamically linked during crop growth.

1.4 Sensitivity analysis of λ, dd, and scf

A sensitivity analysis was conducted to evaluate the effects of λ, dd, and scf on simulated soil temperature.

Because λ is a calibrated model parameter, whereas dd and scf are dynamically calculated variables during model simulation, a one-factor-at-a-time approach was used to separately evaluate the response of the ST submodule to variations in each quantity. When one quantity was varied, the other model conditions were kept unchanged to isolate its effect on simulated soil temperature.

The ranges used in the sensitivity analysis were selected to characterize the structural response of the ST formulation over broad model-relevant intervals and should therefore not be interpreted as the actual temporal ranges observed for each treatment during the field experiment. The selected ranges are summarized in Table S2.

Table S2. Ranges of key quantities used in the sensitivity analysis of the ST submodule

Variable	Physical meaning	Range	Increment
λ	Lag coefficient	0.1–0.9	0.1
dd	Damping depth	500–3500 mm	100 mm
scf	Surface cover factor	0.1–1.0	0.1

To quantify the response of simulated soil temperature to each quantity, the mean soil temperature over the simulation period was calculated as:

$$\overline{T}_{\text{soil}} = \frac{1}{N}\sum_{d=1}^{N} T_{\text{soil}}\,(d) \tag{S4}$$

where N is the number of days in the simulation period and $T_{\text{soil}}(d)$ is the simulated soil temperature on day d.

The maximum variation in mean simulated soil temperature over the investigated range of each quantity, R_T, was then calculated as:

$$R_T = \max\left(\overline{T}_{\text{soil}}\right) - \min\left(\overline{T}_{\text{soil}}\right) \tag{S5}$$

The resulting responses are summarized in Table S3.

Table S3. Responses of mean simulated soil temperature to variations in λ, dd, and scf

Variable	Range	Minimum mean T_{soil} (°C)	Maximum mean T_{soil} (°C)	R_T (°C)
λ	0.1–0.9	14.37	14.83	0.46
dd	500–3500 mm	10.10	15.43	5.33
scf	0.1–1.0	10.09	20.39	10.31

Within the investigated ranges, the three quantities produced different responses in simulated mean soil temperature. Variation in scf produced the largest change, with an R_T of 10.31 °C, indicating that the representation of surface-cover conditions can strongly influence the calculated surface thermal condition and subsequent soil-temperature simulation.

Variation in dd resulted in an R_T of 5.33 °C, indicating that damping depth plays an important role in controlling the attenuation of surface temperature fluctuations through the soil profile.

In comparison, variation in λ from 0.1 to 0.9 resulted in a smaller change in mean soil temperature, with an R_T of 0.46 °C. However, the relatively small variation in mean temperature does not indicate that λ has little influence on soil-temperature dynamics. Because λ primarily determines the contribution of the previous-day soil thermal state to the current-day calculation, its influence is expected to be more evident in temporal persistence and day-to-day temperature variability than in the mean temperature over the complete simulation period.

Importantly, these results describe the response of the ST formulation within the investigated ranges and should not be interpreted as the actual treatment-induced temperature differences observed in the field experiment.

2 Supplementary Methods for the Simplified Model

2.1 Simplified empirical correction of accumulated temperature

For regions where continuous soil temperature observations are unavailable, a simplified empirical approach was explored to approximately account for the effects of straw mulching on crop thermal accumulation without explicitly simulating soil temperature. The method introduces a time-dependent correction into the original AquaCrop air-temperature-based thermal-time calculation while avoiding additional soil thermal properties and heat-transfer parameters.

The empirical temperature correction was expressed as

$$\Delta T_m(d) = \begin{cases} A\exp(-kt), & 0 \le t < T_d \\ 0, & t \ge T_d \end{cases} \tag{S6}$$

where $\Delta T_m(d)$ is the daily empirical temperature correction, A is the initial correction magnitude (°C), k is the decay coefficient (d^{-1}), t is time from the beginning of the correction period, and T_d is a prescribed fixed correction period. Positive and negative values of A represent positive and negative thermal corrections, respectively.

The original air-temperature-based daily growing degree days were calculated as

$$GDD_{\mathrm{air}}(d) = \max[0,\ \min(T_{\mathrm{air}}(d), T_u) - T_b] \tag{S7}$$

where $T_{\mathrm{air}}(d)$ is the daily mean air temperature, and T_b and T_u are the lower and upper temperature thresholds, respectively.

The equivalent temperature after empirical correction was calculated as

$$T_{\mathrm{emp}}(d) = T_{\mathrm{air}}(d) + \Delta T_m(d) \tag{S8}$$

and the corresponding daily effective thermal time was

$$GDD_{\mathrm{emp}}(d) = \max\left[0,\ \min\left(T_{\mathrm{emp}}(d), T_u\right) - T_b\right] \tag{S9}$$

The accumulated effective thermal time up to day n was then calculated as

$$AGDD_{\mathrm{emp}}(n) = \sum_{d=1}^{n} GDD_{\mathrm{emp}}(d) \tag{S10}$$

For comparison, the daily thermal time derived from AquaCrop-ST was calculated as

$$GDD_{\mathrm{ST}}(d) = \max[0,\ \min(T_{\mathrm{ST}}(d), T_u) - T_b] \tag{S11}$$

and

$$AGDD_{\mathrm{ST}}(n) = \sum_{d=1}^{n} GDD_{\mathrm{ST}}(d) \tag{S12}$$

where $T_{\mathrm{ST}}(d)$ is the daily soil temperature simulated by AquaCrop-ST.

2.2 Parameter identification and comparison with AquaCrop-ST

The parameters A and k were preliminarily identified for CS, SS, and WS by searching for the parameter combination that minimized the difference between the equivalent empirical temperature and the corresponding AquaCrop-ST soil temperature. The resulting optimal parameter combinations are summarized in Table S4 and Figure S1.

Table S4. Optimal parameter combinations of the simplified empirical temperature-correction function and associated fitting statistics for the three straw-mulching treatments. The parameters were identified from the present dataset and are intended for exploratory comparison with AquaCrop-ST.

Treatment	A (°C)	k (d^{-1})	RMSE (°C)	Final AGDD difference, Empirical − AquaCrop-ST (°C d)
CS	-2.396	0.3001	1.799	-64.33
SS	1.390	0.0417	1.782	-6.67
WS	-0.229	0	1.848	9.33

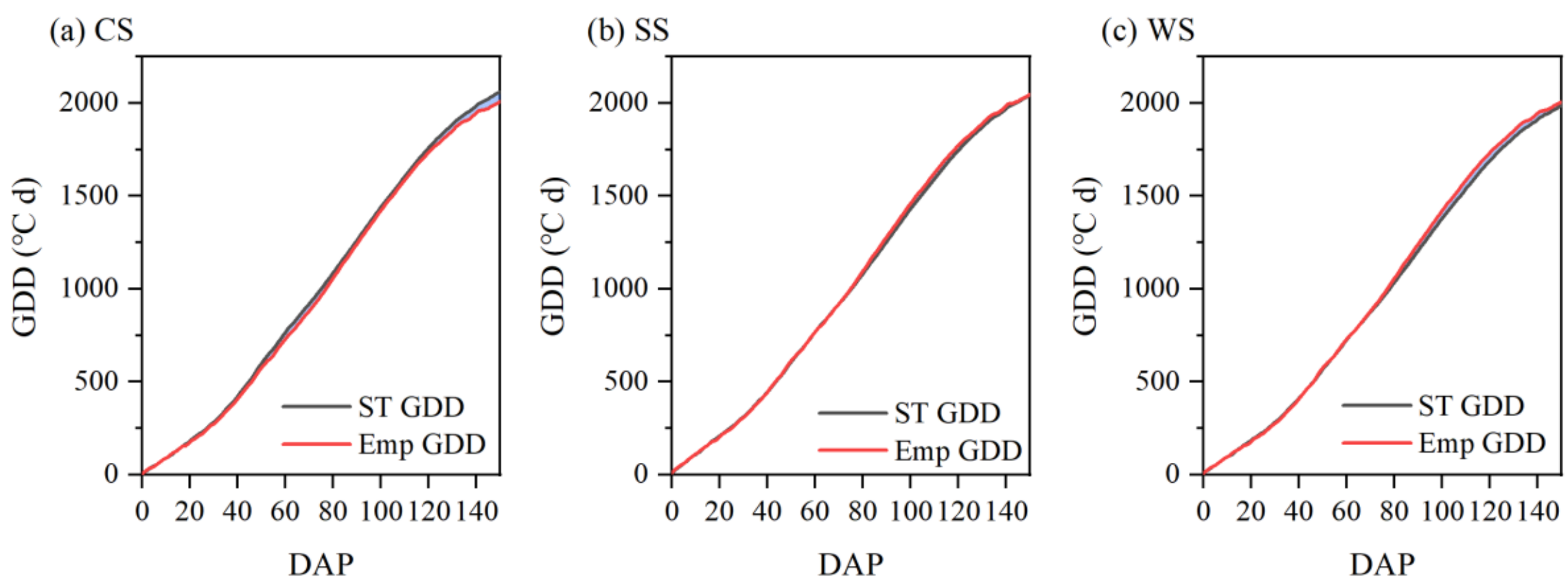

Figure S1. Comparison of accumulated thermal time calculated using the original AquaCrop air-temperature formulation, the simplified empirical correction, and AquaCrop-ST for the CS, SS, and WS treatments. Panels (a), (b), and (c) correspond to the CS, SS, and WS treatments, respectively.

Although the temperature RMSE values were relatively similar among treatments, differences in the final accumulated thermal time were more pronounced, particularly for CS. This is because RMSE reflects the average daily deviation during the correction period, whereas accumulated thermal time integrates these deviations over time; therefore, relatively small but persistent directional errors can result in a larger difference in final AGDD.

The identified parameters also differed substantially among the three mulching treatments. CS was characterized by a relatively large negative initial correction and a rapid decay, whereas SS showed a positive correction with a slower decay. For WS, the optimal decay coefficient approached zero, indicating that an approximately constant correction provided a similar fit to an exponentially decaying response. These differences suggest that the thermal effects of contrasting straw-mulching configurations cannot be fully represented by a single universal exponential correction with common parameters.

The simplified formulation therefore provides a low-complexity approximation of mulch-induced changes in thermal accumulation, particularly when continuous soil temperature information is unavailable. However, the treatment-dependent parameter estimates, weak identifiability of the decay coefficient for some treatments, and the absence of systematic mulch-coverage gradients in the present experiment limit the generalization of the empirical formulation. In addition, the empirical method corrects thermal time directly and does not explicitly simulate the soil temperature process.

AquaCrop-ST, in contrast, provides an explicit representation of soil thermal dynamics and their subsequent influence on crop development. Accordingly, the simplified formulation should presently be regarded as a practical empirical approximation and comparative benchmark rather than a demonstrated substitute for AquaCrop-ST.

2.3 Sensitivity analysis of the simplified formulation

A profile sensitivity analysis was further conducted for k. For each prescribed value of k, the corresponding optimal A was determined and the resulting temperature RMSE was calculated.

Table S5. Profile sensitivity analysis of the decay coefficient k. For each prescribed k, A was re-estimated to minimize the RMSE between the empirical equivalent temperature and AquaCrop-ST soil temperature.

$k\ (d^{-1})$	CS A (°C)	CS RMSE (°C)	SS A (°C)	SS RMSE (°C)	WS A (°C)	WS RMSE (°C)
0.00	0.174	1.867	0.585	1.832	-0.229	1.848
0.01	0.132	1.873	0.792	1.812	-0.251	1.851
0.02	0.053	1.875	1.002	1.796	-0.266	1.853
0.04	-0.191	1.873	1.365	1.782	-0.291	1.856
0.06	-0.491	1.863	1.599	1.789	-0.325	1.856
0.10	-1.063	1.838	1.755	1.823	-0.441	1.856
0.20	-1.968	1.805	1.537	1.882	-0.752	1.852
0.30	-2.396	1.799	1.231	1.904	-0.926	1.851
0.50	-2.688	1.807	0.781	1.918	-1.023	1.852

The sensitivity analysis showed distinct parameter-response patterns among CS, SS, and WS, indicating that k cannot currently be regarded as a universal coefficient across mulch configurations.

Importantly, the present experiment compared different straw-mulching configurations but did not include a systematic gradient of mulch coverage or residue amount. Therefore, the optimal parameter values identified here do not establish a general relationship between A, k, and mulch intensity. The applicability of the simplified function across different coverage levels remains to be evaluated using experiments specifically designed with mulch-amount or coverage gradients.

Overall, the empirical formulation provides a simple means of approximating mulch-induced changes in thermal accumulation when continuous soil temperature information is unavailable. Its comparison with AquaCrop-ST should therefore be regarded as a preliminary methodological exploration rather than a demonstration of general predictive equivalence.

3 Supplementary Figure of CK Calibration

CK processing is used for baseline model calibration, and the canopy cover dynamics observed and simulated under CK are shown in Figure S2.

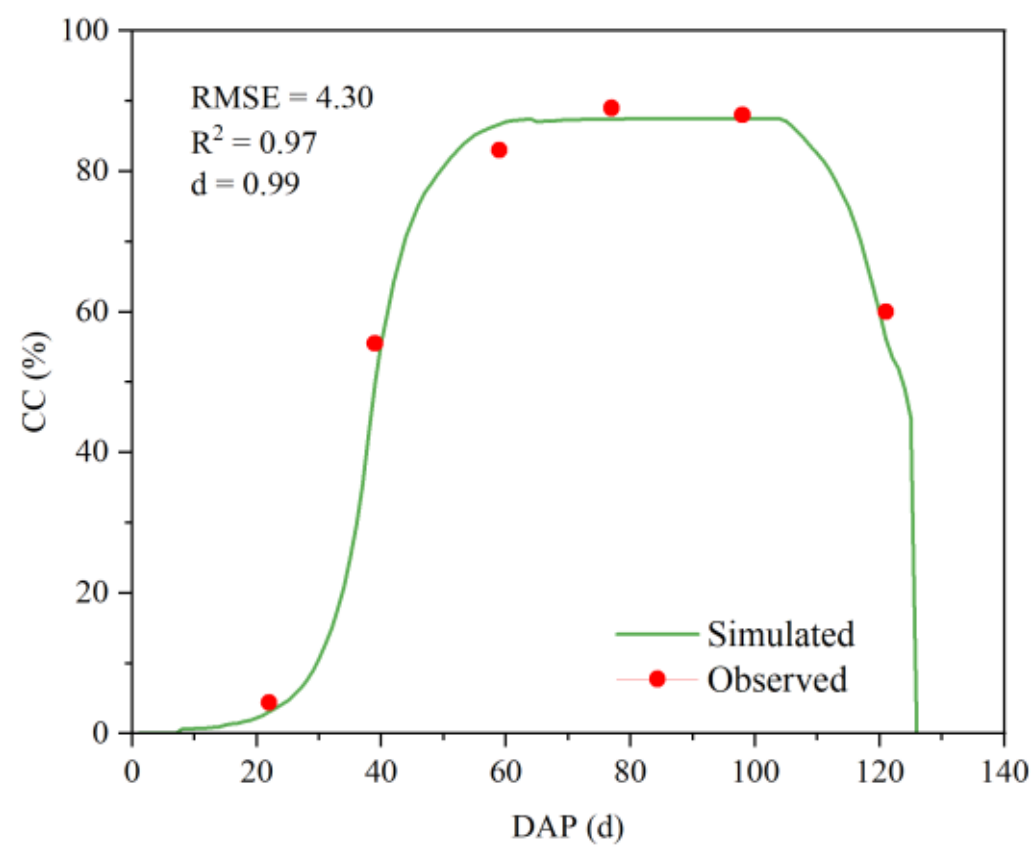

Figure S2. Observed and simulated canopy-cover dynamics for the CK treatment used in baseline model calibration.
